# Altered lipid profiles in the prefrontal cortex are associated with neuroinflammation after severe burn injury

**DOI:** 10.3389/fimmu.2025.1709256

**Published:** 2025-12-01

**Authors:** Sean O’Leary, Anesh Prasai, Ariadna Robledo, Christopher Thang, Ye Wang, Rahul R. Deshpande, William K. Russell, Andrew J. Murton, Steven E. Wolf, Amina El Ayadi

**Affiliations:** 1 Department of Neurosurgery, University of Texas Medical Branch, Galveston, TX, United States; 2 Department of Surgery, University of Texas Medical Branch, Galveston, TX, United States; 3 Department of Medicine, University of Miami, Miami, FL, United States; 4 Department of Dermatology, Massachusetts General Hospital, Harvard Medical School, Boston, MA, United States; 5 Department of Biochemistry and Molecular Biology, University of Texas Medical Branch, Galveston, TX, United States; 6 Sealy Center on Aging, The University of Texas Medical Branch, Galveston, TX, United States; 7 Shriners Children’s Texas, Galveston, TX, United States

**Keywords:** burn injury, lipidomics, neuroinflammation, prefrontal cortex, acipimox, lipolysis, machine learning, cytokines

## Abstract

**Background:**

Severe burn injuries can cause long-term cognitive impairments, potentially driven by lipid-mediated neuroinflammation in the central nervous system (CNS). The disruption of lipid homeostasis may contribute to neuroinflammatory responses, exacerbating neuronal damage. This study investigates whether acipimox, an anti-lipolytic agent, modulates lipid accumulation and neuroinflammation in the prefrontal cortex following severe burns.

**Methods:**

Sprague Dawley rats were randomized into four groups: sham vehicle, sham acipimox, burn vehicle, and burn acipimox. A scald injury covering 40–60% of total body surface area was induced, and rats were treated with acipimox (50 mg/kg/day, intraperitoneally) or vehicle for seven days. Lipidomic analysis assessed alterations in lipid profiles, while machine learning (XGBoost) identified key lipid drivers of burn-induced neuroinflammation. Additionally, mRNA expression of inflammatory markers, including interleukin-1β (IL-1β), nuclear factor kappa-light-chain-enhancer of activated B cells (NF-κB), and toll-like receptor 4 (TLR4), was quantified to evaluate neuroinflammatory responses. Cytokine–lipid correlations were also examined using Spearman analysis.

**Results:**

Lipidomic analysis identified significant alterations in a subset of the 21 lipid classes analyzed, particularly long-chain and very-long-chain fatty acids, including lysophosphatidylethanolamines, lysophosphatidylcholines, phosphatidylglycerols, phosphatidylethanolamines, and triacylglycerols (*p* < 0.05). Machine learning (XGBoost) identified these lipids as significantly modulated with burn injury (AUC > 0.80). Acipimox treatment reduced lipid accumulation, restoring levels to sham values. Furthermore, mRNA analysis showed group differences in IL-1β (overall ANOVA p = 0.030), with significant pairwise difference observed for burn-vehicle vs sham-acipimox. Acipimox also modulated NF-κB and TLR4 expression, indicating attenuation of inflammatory signaling. IL-1β and LPL positively correlated with lipid classes elevated by burn and reversed by acipimox, while IL-6, TNF-α, NF-κB, and TLR4 showed predominantly negative associations.

**Discussion:**

These findings suggest that severe burns induce significant lipid dysregulation in the CNS, contributing to neuroinflammation and potential cognitive impairment. By targeting lipolysis, acipimox mitigates lipid accumulation, suppresses inflammatory pathways, and normalizes lipid levels, highlighting a potential therapeutic mechanism.

**Conclusion:**

This study establishes a mechanistic link between elevated lipolysis and CNS inflammation following severe burns. Acipimox effectively modulates lipid profiles and reduces neuroinflammation, underscoring its potential for managing burn-induced neurological complications. Further studies are needed to validate these findings and explore clinical applications.

## Introduction

Cognitive impairments are long-term outcomes that have been reported following severe trauma such as burns (1). These may manifest as memory loss, amnesia, dementia, depression, anxiety, post-traumatic stress disorder (PTSD), hallucinations, and delirium (1–7). Patients hospitalized for burns are more than twice as likely to be admitted for conditions affecting the nervous system compared to those without burn injuries (8–10). Given the increased risk of central nervous system (CNS) complications following severe burns, addressing the effect of burn on the brain is crucial for improving the long-term prognosis of severe burn patients (8). Severe burns are associated with a prolonged hypermetabolic response (10, 11) characterized by increased proteolysis and lipolysis and loss of lean body mass, a response driven by persistent catecholamine production. Clinical studies into post-burn lipolysis have primarily focused on free fatty acids, plasma triglycerides, and cholesterol, offering a limited glimpse into the effects of post-burn metabolic dysfunction on the CNS (12). Cutaneous burn injury was shown to induce neuroinflammation and activate astrocytes in the hippocampus (13) by a still-unknown mechanism. Increased levels of inflammatory cytokines in the circulation and the brain have been linked to disruption of the blood-brain barrier (BBB) (14). Increased peripheral levels of fatty acids may also induce the accumulation of advanced glycation end-products (AGEs), resulting from unspecific and uncontrolled reactions between proteins or lipids and carbohydrates, leading to AGE-mediated disruption of the BBB (15). Recent plasma lipidomic analyses further revealed that burn injury induces systemic lipidomic alterations that persist throughout early life, suggesting durable metabolic reprogramming that may influence nervous system outcomes (16). These findings underscore the need to investigate lipid-mediated mechanisms linking peripheral burn injury to central neuroinflammation.

In this study, we sought to determine the effects of severe burn on rodent prefrontal cortex lipid profile and to characterize the neuroinflammatory response driven by increased lipolysis post-burn. To depict the effects of lipid accumulation on neuroinflammation in a rodent model of severe burns, we utilize acipimox, a niacin derivative with potent anti-lipolytic activity. Acipimox-mediated inhibition of lipolysis and adipose browning is associated with a beneficial metabolic phenotype (17). Acipimox acts as a ligand to G-protein-coupled receptor GPR109A to modify lipidomic profiles in humans, used therapeutically for its elevation of HDL while decreasing VLDL and LDL (18). Acipimox administration for 12 weeks was shown to decrease triglycerides in patients with Type 2 diabetes by 19% (19). Lipidomic studies did not show major lipid species differences between humans and rats, making the rat model ideal for lipid-associated burn-induced neuroinflammation resulting from increased lipid levels (20, 21). To determine the role of lipids in burn-induced neuroinflammation, we utilized an established rat model of severe burns treated with acipimox daily for one week. This approach allowed us to systematically evaluate the effectiveness of acipimox in mitigating dyslipidemia-driven inflammation associated with burn injury.

## Methods

### Animal experiments

All animal studies were performed under UTMB-approved protocol #1812094. Male Sprague Dawley rats were obtained from Charles River Laboratories (Wilmington, MA, USA) and acclimated for one week in a controlled vivarium environment prior to experimentation. Males were selected because clinical burn cohorts show greater inflammatory and hypermetabolic responses, more severe clinical courses, and longer hospital stays than female (22, 23). Animals were then randomly assigned to receive either a full-thickness scald burn covering 40–60% of total body surface area (TBSA) or a sham procedure. For the burn procedure, rats received buprenorphine (0.05 mg/kg SC) and isoflurane anesthesia. The animals were shaved before immersion in 96-98°C water for 10s for the dorsum 2s for the abdomen, using a custom mold. Lactated Ringer’s (30 ml/kg IP) was given for resuscitation. We did not observe a weight increase with Lactated Ringers resuscitation. The Sham group received lukewarm water immersion without fluid resuscitation. To measure chronic muscle protein synthesis, all rats received 70% heavy water (10 ml/kg IP) post-procedure, with drinking water supplemented with 2% heavy water thereafter. Both groups had a mortality rate of zero.

After the burn procedure, animals were randomized to one of 4 groups: sham vehicle (n = 8), sham acipimox (n = 8), burn vehicle (n = 8), and burn acipimox (n = 7). Animals received either vehicle (PBS) control or acipimox (50 mg/kg/day (17), i.p.) for 7 days.

**Figure 1:**
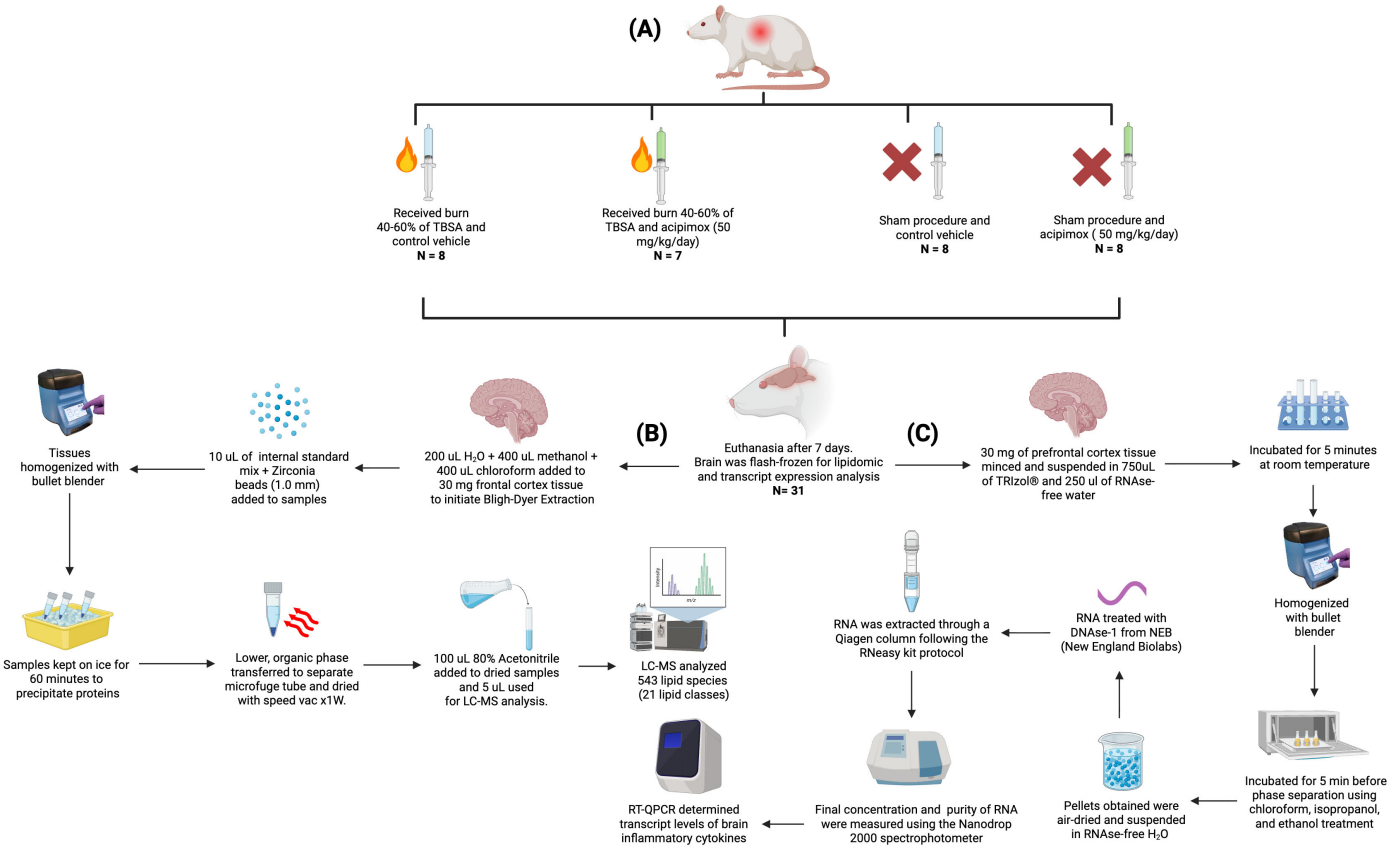
Experimental workflow for animal procedures, lipid quantification, and RT-qPCR. **(A)** Experimental design and animal treatment groups, including burn injury induction, sham procedures, and acipimox or vehicle administration. **(B)** Lipid extraction and targeted lipidomic analysis pipeline using Bligh–Dyer extraction, LC-MS/MS profiling, and normalization via internal standards. **(C)** RNA extraction and RT-qPCR workflow for quantifying inflammatory cytokine transcripts from prefrontal cortex tissue. Figure created with Biorender.com.

Since the same animals were used in muscle metabolism studies, they were subdivided to receive either Leucine (1.35 g/kg oral) + insulin (100 mU Actrapid IP) or PBS on day **7**, and after a 6-hour fast. Rats were euthanized using CO_2_ followed by cervical dislocation as approved in the IACUC protocol. All rats received 2-deoxyglucose (0.5 g/kg IP) and 2H5-phenylalanine (50 mg/kg IV) within 60 minutes of euthanasia. Blood, muscle, and brain tissue were quickly collected and flash-frozen. Brain tissues were processed for lipidomic and transcript expression analysis. As illustrated in **Figure 1**, tissues were homogenized using a bullet blender (Next Advance, Averill Park, NY, USA). A total of 543 lipid species across 21 lipid classes were measured using LC-MS. The data were normalized using isotopically labeled internal standards added to each sample depending on their respective tissue weights. RT-QPCR was used to determine the transcript levels of brain inflammatory cytokines (**Figure 1A**).

### Broad lipid quantification using high throughput targeted lipidomics

Bligh-Dyer Extraction of lipids was performed on brain tissues. 200 µL of water, 400 µL of methanol, and 400 µL of chloroform were added to 30 mg prefrontal cortex tissue. 10 µL of the internal standard mix EquiSPLASH™ LIPIDOMIX^®^ Quantitative Mass Spec Internal Standard (Avanti Polar Lipids, Alabaster, AL, USA; cat. no. 330731) was added to the samples and Zirconia beads (1.0 mm) (Next Advance, Averill Park, NY, USA) were added to each tube, and tissues were homogenized using a bullet blender (Next Advance, Averill Park, NY, USA). Samples were kept on ice for 60 minutes to precipitate proteins. The lower, organic phase was carefully transferred to a separate microfuge tube and dried using an evaporator (SpeedVac, Thermofisher Scientific). 100 µL of 80% Acetonitrile was added to the dried samples and 5 µL of the reconstituted sample was used for LC-MS analysis. LC-MS analysis was performed utilizing the HILIC column for lipid species. The data was analyzed using Multiquant MD Software (SCIEX). The peak areas obtained were normalized to the peak areas of internal standards to obtain area ratios. Each area ratio was further normalized to the weight of respective tissues to account for sample differences. The normalized area ratios were used for statistical analysis to compare treatment groups (**Figure 1B**).

### RNA extraction and RT-QPCR

30 mg of rat prefrontal cortex tissue was minced and suspended in 750uL of TRIzol^®^ (Ambion, Foster City, CA) plus 250 µL of RNAse-free water and incubated for 5 minutes at room temperature before homogenization using the bullet blender homogenizer and zirconium beads (Next Advance, Averill Park, NY, USA). Samples were then incubated for 5 min before phase separation using chloroform, isopropanol, and ethanol treatment as per the manufacturer’s instructions. The pellet obtained was air-dried and suspended in RNAse-free water. The RNA was then treated with DNAse-1 from NEB (New England Biolabs, MA) and extracted through a Qiagen column following the Rneasy kit protocol (Qiagen, Valencia, CA). The final concentration and the purity of RNA were measured using the Nanodrop 2000 spectrophotometer (Thermoscientific, Waltham, MA) and by running the samples in agarose gel respectively.

RT-QPCR was used to determine the transcript levels of brain inflammatory cytokines.

Synthesis of cDNA was performed with the iScript™ cDNA Synthesis Kit (Bio-Rad Laboratories, Hercules, CA). Real-time PCR was performed using the Applied Biosystems^®^ StepOnePlus™. PCR primer sets are listed in **Supplementary Table 1**. Cyc-A served as an internal control (**Figure 1C**).

### Mass spectrometry

LC-MS/MS analysis was performed using a 1290 Infinity UHPLC System (Agilent) coupled to a Turbo V electrospray ionization source and a Qtrap 6500 mass spectrometer (SCIEX). The samples were separated in HILIC mode using a polymer-based X-Bridge amide 4 µm, 150 × 4.6 mm column (Waters). The LC conditions were as follows Mobile phase A: 95% ACN with 10 mM NH 4 CH 3 CO 2 and 5% H 2 O. Mobile phase B: 50% ACN with 50% H 2 O and 10 mM NH 4 CH 3 CO 2. The gradient was: 0 min 99% A, 0–6 min 94% A, 6–10 min 75% A, 10–11 min 2% A, 11–13 min 0% A, 18–19 min 99% A, 19–24 min 99% A, 24 min stop. The flow rate was 700 µl/min, column temperature 35°C and the injection volume is 10 µl. The MS/MS detection is performed using electrospray ionization (ESI) and by scheduled multiple reaction monitoring (MRM). All 1150 lipids and internal standards are targeted in a single injection using both negative and positive modes with rapid polarity switching and scheduled MRM algorithm. The ESI source conditions are set as follows: electrospray voltage of −4500 V for negative mode and 5500 V for positive mode, source temperature of 500°C, curtain gas of 35, ion source gas 1 and gas 2 of 55 and 50 psi, respectively.

### Data processing and normalization

Data analysis was first passed through relative standard deviation (RSD) filtering within quality control (QC) samples (MetaboAnalyst 6.0). Features displaying an RSD greater than 25% were considered unreliable and subsequently filtered from the dataset. Secondly, Interquartile Range (IQR) filtering was employed to remove features falling outside of the 40% IQR, this reduced the impact of outlier data points. Data was transformed through base-10 logarithmic transformation as well as mean-centered and standard deviation scaled. For each variable x, the scaled x’ was calculated as x’ = x – mean(x)/std(x). Results of normalization are shown in **Supplementary Figure 1**. Data before normalization is provided in **Supplementary Data Sheet 1**, and after normalization in **Supplementary Data Sheet 2**.

### Clustering and discriminant data analysis

Hierarchical Clustering was performed in RStudio using the cluster package to visualize group variation for each lipid species. One-way analysis of Variance (ANOVA) was performed, and resultant p-values were adjusted using the Bonferroni-Holm method (24). The top 50 lipids were then clustered with a Euclidean distance matrix computed, which was then subjected to hierarchical clustering using Euclidean distance and Ward’s minimum variance method (25). The heatmap.2 function was used for plotting and visualization. Partial least squares-discriminant analysis (PLS-DA) in MetaboAnalyst (version 6.0) was used for identifying group separation. Reported values for PLS-DA include R2, Q2, and accuracy results for 5-fold cross-validation, VIP feature importance scores for top 20 features, and results of permutation test with separation of distance (B/W) set as test statistic and 100 permutations to assess the hypothesis of no effect with significance determined by p < 0.05. PLS-DA results were displayed in 2D and 3D score plots.

### Lipid group comparison data analysis

Lipid classes were then analyzed using RStudio with ggplot2 for visualization, with preliminary group variation determined through one-way ANOVA tests to discern significant (p < 0.05) group variations from the 21 lipid categories following Bonferroni-Holm multiple p-value correction: Cholesteryl Ester (CE), Ceramide (CER), Diacylglycerol (DAG), Dihydroceramide (DCER), Free Fatty Acid (FFA), Lysophosphatidylcholine (LPC), Lysophosphatidylethanolamine (LPE), Lysophosphatidylglycerol (LPG), Lysophosphatidylinositol (LPI), Lysophosphatidylserine (LPS), Monoacylglycerol (MAG), Phosphatidylcholine (PC), Phosphatidylethanolamine (PE), Phosphatidylethanolamine O-alkyl (PE-O), Phosphatidylethanolamine plasmalogen (PE-P), Phosphatidylglycerol (PG), Phosphatidylinositol (PI), Phosphatidylserine (PS), Sphingomyelin (SM), Triacylglycerol (TAG), and Polyunsaturated Fatty Acid (PUFA). Lipid species analyzed, along with associated lipid class assignment, are provided in **Supplementary Data Sheet 3**. Significant groups were then further analyzed using Tukey’s *post hoc* test to determine all significant (p < 0.05) pairwise comparisons found between the experimental groups. For Tukey’s *post hoc* tests, mean and standard error of the mean (SEM) were reported for the analyzed normalized area ratios.

### Machine learning for biomarker discovery

To further delineate significant group variation, XGBoost machine learning modeling was then employed in RStudio utilizing the caret, xgboost, and pROC packages, with ggplot2 and ggrepel for visualization. XGBoost as a machine learning technique has empirically been used for lipidomic analysis, and leverages ensemble machine learning to enhance prediction and accuracy (26). XGBoost leverages an ensemble of decision trees through gradient boosting, allowing sequential training that minimizes the loss function at each step by adjusting for errors from prior trees (20). This process enables XGBoost to achieve high predictive accuracy by iteratively improving model performance on gradient statistics (20).

We implemented supervised classification with XGBoost in RStudio using the caret interface to xgboost for model training and tuning and pROC for performance assessment, with ggplot2/ggrepel for visualization. Analyses were performed on the normalized dataset described above. For each pairwise group comparison among the four cohorts (burn-vehicle, burn-acipimox, sham-vehicle, sham-acipimox), we constructed binary classifiers using the relevant lipid features for that contrast. Within each contrast, centering, scaling, and model fitting were performed inside a 5-fold, stratified cross-validation routine implemented by caret::train to prevent information leakage, with set.seed(1234) for reproducibility. When a marked class imbalance was detected (minority class <20% of samples), we applied caret::upSample prior to training within the resampling loop. Hyperparameters for xgbTree were selected by caret’s internal grid search optimizing the cross-validated area under the receiver operating characteristic curve (AUC). ROC curves and AUCs were computed from held-out fold predictions using pROC.

To interpret models, variable importance was extracted via caret::varImp (XGBoost gain), computed within folds and then aggregated across folds. Importance scores were min–max normalized to 0–100 to provide a standardized importance scale. For each comparison, the lipid class achieving the highest cross-validated AUC and its constituent species with non-zero importance were designated as “modulatory” features for that comparison. We then retrained XGBoost models using only this modulatory subset for each contrast under the same 5-fold cross-validation scheme to verify separability. Finally, to relate model-selected features back to group biology, we subjected the modulatory lipid subset to one-way ANOVA with Tukey’s *post hoc* tests on the normalized area ratios (mean ± SEM reported; α = 0.05).

### mRNA expression data analysis

Relative mRNA expression levels of interleukin-1 beta (IL-1β), interleukin-6 (IL-6), nuclear factor kappa-light-chain-enhancer of activated B cells (NF-κB), toll-like receptor 4 (TLR4), tumor necrosis factor alpha (TNF-α), and lipoprotein lipase (LPL) were quantified using the 2^ (-ΔΔC(T)) method. The Ct values of the target genes in each sample were normalized to the Ct value of a housekeeping gene (Cyc-A) from the same sample to obtain the ΔCt value. The ΔΔCt value was then calculated by subtracting the ΔCt value of the control group (sham vehicle) from the ΔCt value of the experimental groups (burn acipimox, burn vehicle, and sham acipimox). The fold change in gene expression was calculated as 2^(-ΔΔC(T)). Values for groups were reported as mean ± SEM. ANOVA testing with Tukey’s *post hoc* analysis for pairwise group comparisons between all groups was performed. A p-value <0.05 was considered statistically significant.

We additionally quantified correlations between lipid levels and cytokine expression using per-sample data at the lipid class level. Cytokine measurements (LPL, TLR-4, TNF-α, NF-κB, IL-6, IL-1β) were aligned to each lipid class sample within its treatment group. For each cytokine, we computed Spearman rank correlations (ρ) between class abundance and cytokine level with pairwise group contrasts across all combinations of S_V, B_V, S_A, and B_A. Nominal p-values were obtained from cor.test, and within each cytokine×contrast we applied Benjamini–Hochberg FDR adjustment. q < 0.05 was the *a priori* significance threshold.

### Sample size sensitivity analysis

This discovery-oriented study complied with ARRIVE/3Rs constraints and did not pre-specify a single primary endpoint. We therefore conducted a prospective-style sensitivity analysis for the actual group sizes (sham-vehicle n=8; sham-acipimox n=8; burn-vehicle n=8; burn-acipimox n=7) at α=0.05. For a fixed-effects one-way ANOVA with four groups (df1 = 3, df2 = 27), the design provides 80% power to detect an omnibus effect size of Cohen’s f = 0.64 (partial η² = 0.29), consistent with a large factor effect. Expressed in pairwise terms, Tukey-adjusted comparisons between groups of size 7–8 provide 80% power for standardized mean differences of d = 1.75–1.80. Calculations used the noncentral F distribution for the omnibus test and standard approximations for Tukey-adjusted pairwise contrasts.

## Results

### Multivariate clustering and discriminant analysis of lipid variability reveals differences across experimental groups

**Figure 2:**
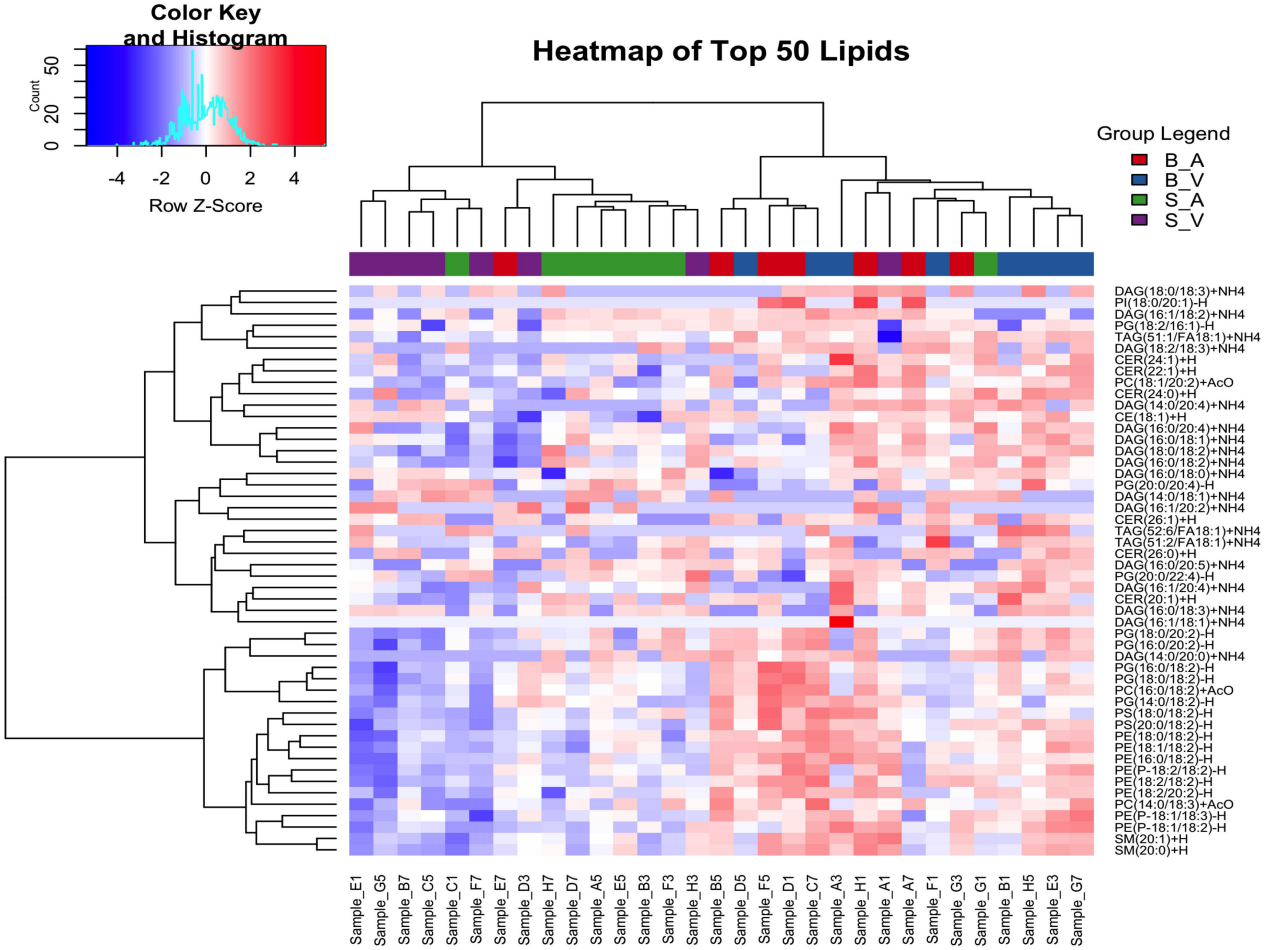
Hierarchical clustering analysis of the top 50 changing lipids identified by ANOVA across experimental groups. The color key and histogram (top left) display clustering degree, and group legends (top right) denote experimental groups. B_V, Burn Vehicle; B_A, Burn Acipimox; S_A, Sham Acipimox; S_V, Sham Vehicle.

Hierarchical clustering analysis was performed for the top 50 lipids selected due to variance between groups as determined by ANOVA. All lipid species had non-significant ANOVA variation between groups following correction for multiple testing using the false discovery rate method (p > 0.05), details provided in **Supplementary Data Sheet 4**. Clustering was done using Euclidean distance and Ward’s method, resulting in four distinct clusters: Cluster 1 included a mix of lipid classes, with 3 DAGs, 1 PG, 1 PI, and 1 TAG lipid. Cluster 2 contained 1 DAG, 2 PC, 8 PE, 5 PG, 2 PS, and 2 SM lipids. Cluster 3 featured 3 CER, 7 DAG, 2 PG, and 2 TAG lipids. Cluster 4 was composed of 1 CE, 3 CER, 5 DAG, and 1 PC lipid. Full cluster details are provided in **Supplementary Table 2**. The separation between groups can be seen with the burn acipimox and burn vehicle groups experiencing high levels of clustering when compared to the sham vehicle and sham acipimox cohorts, as represented in **Figure 2**.

**Figure 3:**
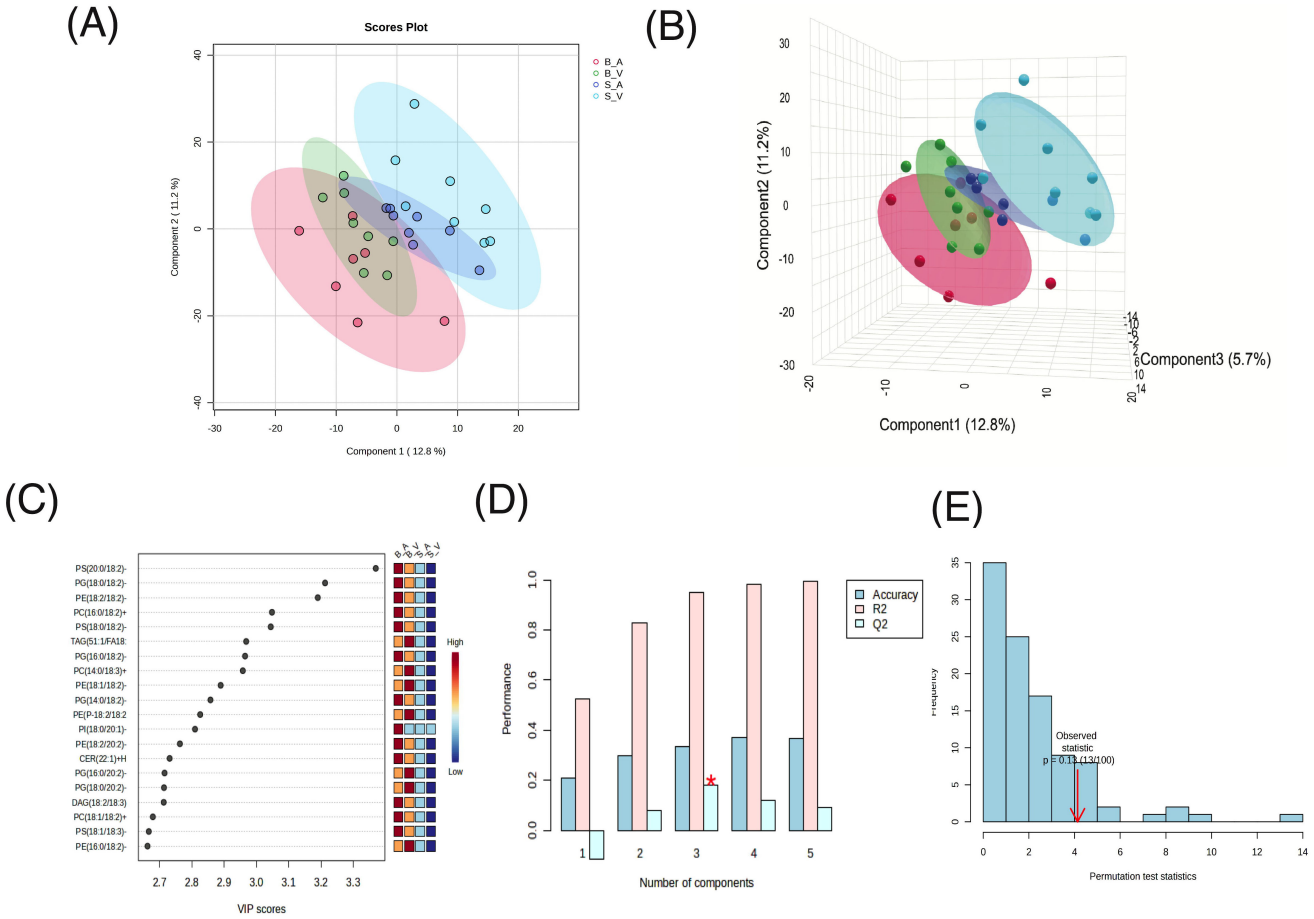
Partial Least Squares-Discriminant Analysis (PLS-DA) results. **(A)** 2D Scores plot, **(B)** 3D Scores plot, **(C)**, Variable Importance in Projection (VIP) feature importance scores for the top 20 features in component 1, **(D)** R², Q², and accuracy results from 5-fold cross-validation, with peak accuracy marked by a red asterisk (*), and **(E)** permutation test results to assess the hypothesis of no effect.

In the Partial Least Squares Discriminant Analysis (PLS-DA) conducted, the largest separation was achieved between the burn acipimox and sham vehicle group, with a smaller yet still present degree of separation achieved between burn vehicle and sham acipimox with 2 components (**Figure 3A**) and 3 components (**Figure 3B**). PLS-DA coefficients, loadings, and scores are provided in **Supplementary Data Sheets 5**–**7** respectively. Regarding the determination coefficient (R2) values, the first through fifth components yielded R2 values of 0.52406, 0.82811, 0.95, 0.98108, and 0.99647, respectively. Meanwhile, the predictive ability (Q2) of the model varied across components. The Q2 values for one to five components were -0.11444, 0.07808, 0.17967, 0.11805, and 0.092439, respectively. It is pertinent to highlight that the negative Q2 value for the single component model suggests potential overfitting (**Figure 3C**). Cross validation results are provided in **Supplementary Table 3**. Variable Importance Scores (VIP) for each of the top 8 components consistently showed PS(20:0/18:2)-H as the most influential to class separation, followed by PG(18:0/18:2)-H and PE(18:2/18:2)-H (**Figure 3D**). All VIP data results are provided in **Supplementary Data Sheet 8**. Utilizing 5-fold cross-validation, the observed accuracy for the PLS-DA model with 1 to 5 components was 0.20857, 0.29905, 0.33238, 0.37238, and 0.36762 respectively. Additionally, a permutation test was performed to assess the hypothesis of no effect. 100 permutations for prediction of accuracy during training were used, with a resultant derived empirical p-value of p = 0.13 indicating the results from PLS-DA were not significant (**Figure 3E**).

### Significant lipid class alterations follow burn injury and acipimox treatment

**Figure 4:**
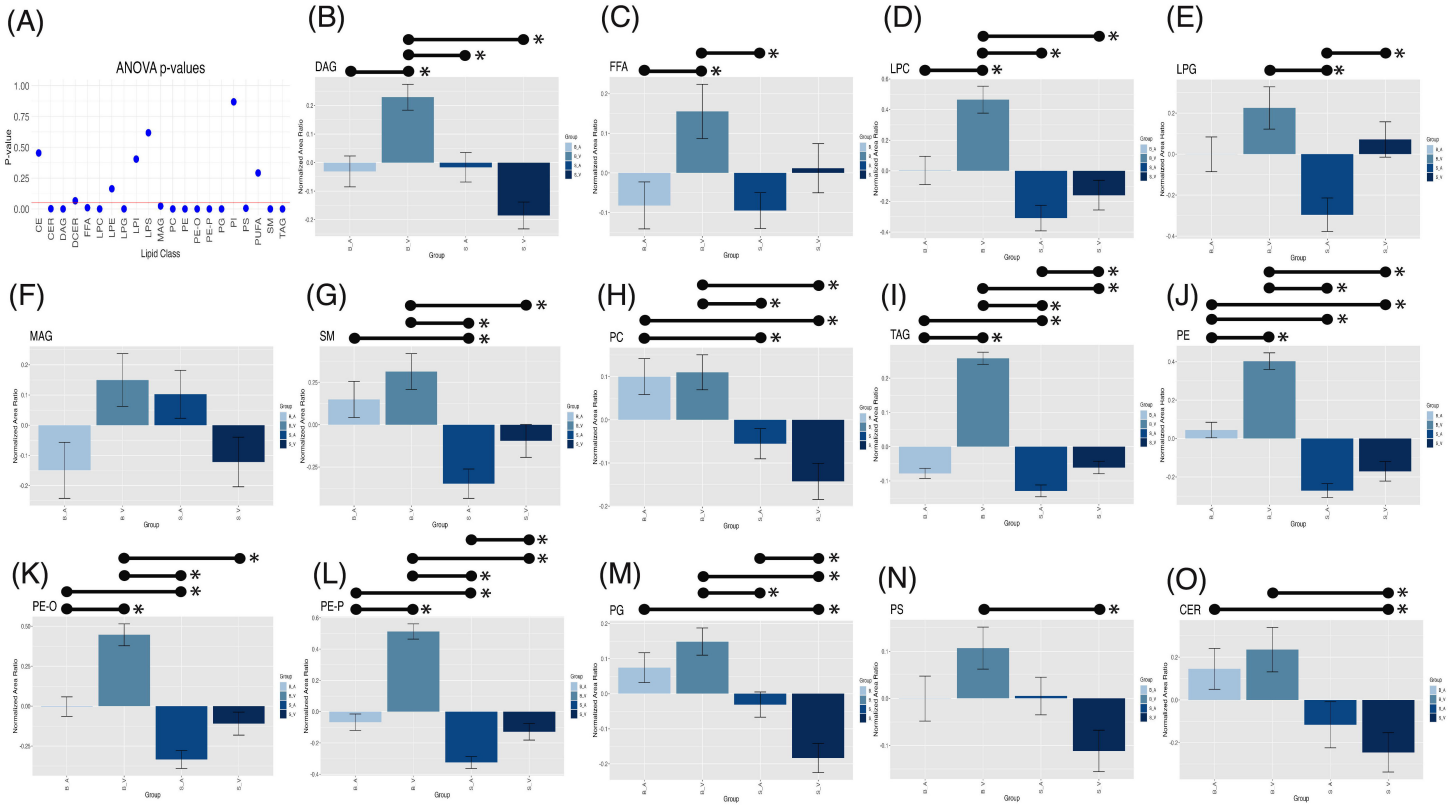
One-way ANOVA p-values **(A)** with a red horizontal line indicating the 0.05 alpha threshold. Tukey’s *post-hoc* pairwise comparisons for lipid classes across experimental groups for DAG **(B)**, FFA **(C)**, LPC **(D)**, LPG **(E)**, MAG **(F)**, SM **(G)**, PC **(H)**, TAG **(I)**, PE **(J)**, PE-O **(K)**, PE-P **(L)**, PG **(M)**, PS **(N)**, and CER **(O)**. Error bars represent standard error of the mean (SEM), with significant (p < 0.05) pairwise comparisons shown by lines spanning significant groups and asterisks (*). B_V, Burn Vehicle; B_A, Burn Acipimox; S_A, Sham Acipimox; S_V, Sham Vehicle; DAG, Diacylglycerol; FFA, Free Fatty Acid; LPC, Lysophosphatidylcholine; LPG, Lysophosphatidylglycerol; MAG, Monoacylglycerol; SM, Sphingomyelin; PC, Phosphatidylcholine; TAG, Triacylglycerol; PE, Phosphatidylethanolamine; PE-O, Phosphatidylethanolamine O-alkyl; PE-P, Phosphatidylethanolamine plasmalogen; PG, Phosphatidylglycerol; PS, Phosphatidylserine; CER, Ceramide.

One-way Analysis of Variance (ANOVA) test performed within lipid classes, using a stringent Bonferroni-Holm multiple-testing correction, revealed significant group variation for multiple lipid classes, shown in **Figure 4A**, including CER (*p* = 0.002), DAG (*p* < 0.001), FFA (*p* = 0.010), LPC (*p* < 0.001), LPG (*p* < 0.001), MAG (*p* = 0.024), PC (*p* < 0.001), PE (*p* < 0.001), PE-O (*p* < 0.001), PE-P (*p* < 0.001), PG (*p* < 0.001), PS (*p* = 0.005), SM (*p* < 0.001), and TAG (*p* < 0.001). All other groups showed no significance in one-way ANOVA variation (*p* > 0.05, see **Supplementary Table 4**). Tukey’s *post-hoc* comparisons identified significant pairwise differences among groups, as detailed in **Supplementary Table 5** and **Figures 4B–O**.

In the burn vehicle *vs.* burn acipimox comparison, which allows for evaluation of the therapeutic effect of acipimox, several lipid classes displayed significantly higher normalized area ratios in the burn vehicle group. DAGs showed a substantial increase (*p* = 0.002, difference = 0.242, 95% CI: 0.067 to 0.417, **Figure 4B**), as did FFAs (*p* = 0.036, difference = 0.220, 95% CI: 0.010 to 0.431, **Figure 4C**) and LPCs (*p* < 0.001, difference = 0.493, 95% CI: 0.172 to 0.813, **Figure 4D**). PEs were also elevated in the burn vehicle group (*p* < 0.001, difference = 0.376, 95% CI: 0.224 to 0.529, **Figure 4J**), along with both ether-linked PE forms: PE-O (*p* < 0.001, difference = 0.448, 95% CI: 0.217 to 0.678, **Figure 4K**) and PE-P (*p* < 0.001, difference = 0.571, 95% CI: 0.401 to 0.742, **Figure 4L**). Additionally, TAGs were significantly higher in the burn vehicle group compared to burn acipimox (*p* < 0.001, difference = 0.293, 95% CI: 0.232 to 0.354, **Figure 4I**). Notably, no lipid classes were significantly elevated in the burn acipimox group compared to the burn vehicle group. These findings indicate that acipimox effectively reduced elevated lipid levels in neuronal tissue following burn injury.

In the sham vehicle *vs.* burn vehicle comparison, which allows for an assessment of burn injury alone, several lipid classes were significantly lower in the sham vehicle group. Specifically, CERs were reduced (*p* = 0.003, difference = -0.481, 95% CI: -0.837 to -0.124, **Figure 4O**), as were DAGs (*p* < 0.001, difference = -0.415, 95% CI: -0.590 to -0.240, **Figure 4B**) and LPCs (*p* < 0.001, difference = -0.624, 95% CI: -0.945 to -0.304, **Figure 4D**). Additionally, PEs showed a significant decrease (*p* < 0.001, difference = -0.573, 95% CI: -0.726 to -0.420, **Figure 4J**), as did both ether-linked forms: PE-O (*p* < 0.001, difference = -0.555, 95% CI: -0.786 to -0.325, **Figure 4K**) and PE-P (*p* < 0.001, difference = -0.642, 95% CI: -0.812 to -0.471, **Figure 4L**). Other lipids with significant decreases in the sham vehicle group included PCs (*p* < 0.001, difference = -0.253, 95% CI: -0.393 to -0.112, **Figure 4H**), PGs (*p* < 0.001, difference = -0.332, 95% CI: -0.472 to -0.192, **Figure 4M**), PS (*p* = 0.002, difference = -0.218, 95% CI: -0.374 to -0.063, **Figure 4N**), SM (*p* = 0.015, difference = -0.409, 95% CI: -0.760 to -0.058, **Figure 4G**), and TAGs (*p* < 0.001, difference = -0.319, 95% CI: -0.380 to -0.258, **Figure 4I**). Notably, no lipid classes were significantly elevated in the sham vehicle group compared to the burn vehicle group. These findings demonstrate that burn injury results in significantly elevated lipid levels in neuronal tissue.

In the sham vehicle *vs.* sham acipimox comparison, which isolates baseline effects of acipimox independent of injury, fewer significant differences were observed similar to the sham vehicle *vs.* burn acipimox group. TAG levels showed an increase in the sham vehicle group (*p* = 0.021, difference = 0.068, 95% CI: 0.007 to 0.129, **Figure 4I**), and LPG levels were also higher (*p* = 0.016, difference = 0.367, 95% CI: 0.049 to 0.685, **Figure 4E**). Additionally, PE-O exhibited a significant increase in the sham vehicle group (*p* = 0.017, difference = 0.196, 95% CI: 0.025 to 0.367, **Figure 4K**), while PG levels were lower in the sham vehicle group (*p* = 0.027, difference = -0.152, 95% CI: -0.293 to -0.012, **Figure 4M**). These findings indicate that even in the absence of burn injury, acipimox treatment modulates lipid levels.

### Machine learning identifies modulatory lipid biomarkers in burn injury and acipimox treatment

**Figure 5:**
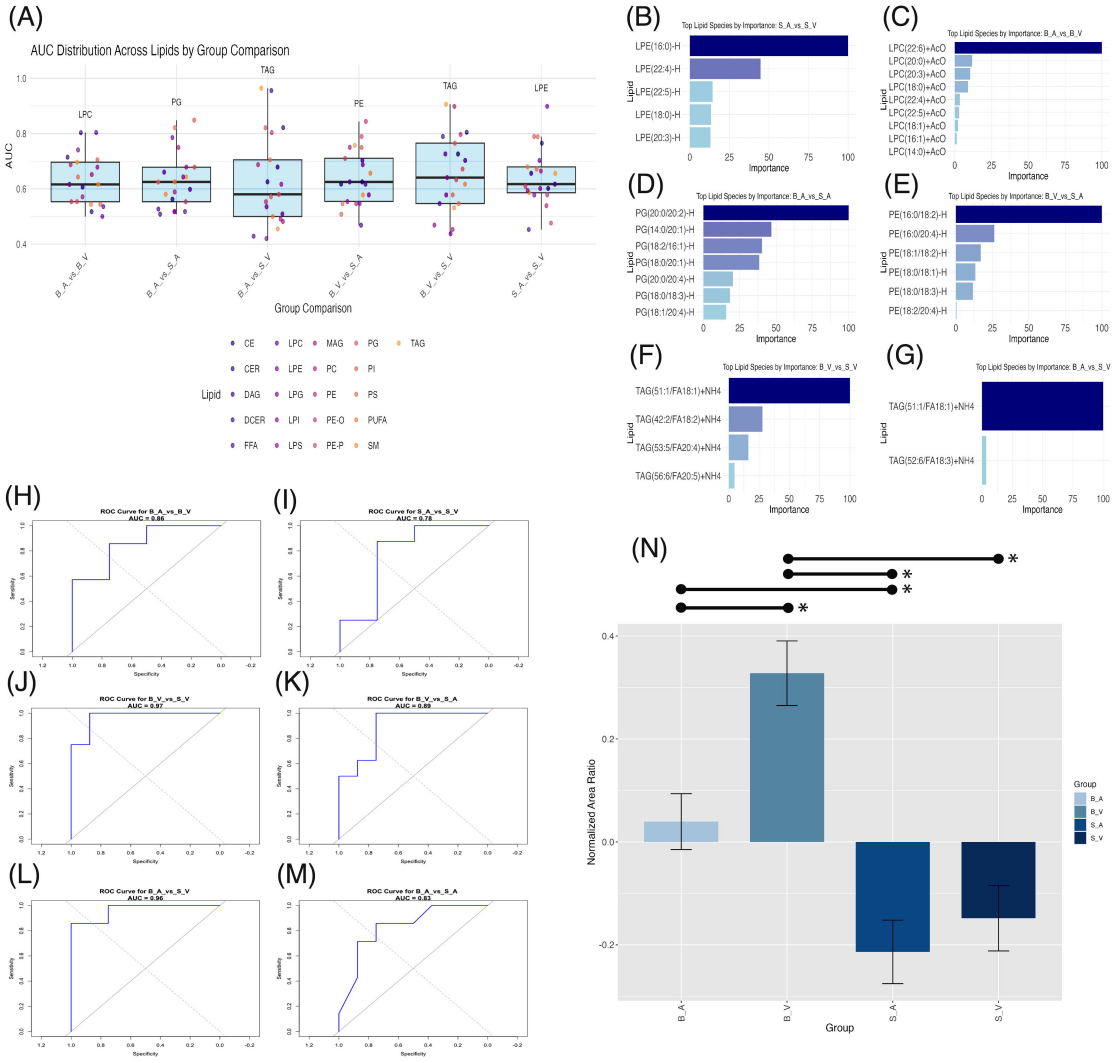
Machine learning lipid biomarker discovery across group comparisons. **(A)** Box plots of area under the curve (AUC) distribution across lipid classes with significant ANOVA results per group comparison. The highest AUC lipid class is labeled above each plot. **(B–G)** Bar plots of importance weights for lipid species within lipid classes contributing significantly (importance > 0) to XGBoost model classification. **(H–M)** Receiver Operating Characteristic (ROC) curves for XGBoost models with AUC values for each comparison. **(N)** Bar plot of normalized area ratios for the modulatory lipid group, with error bars representing SEM and significant (p < 0.05) pairwise comparisons shown by lines spanning significant groups and asterisks (*). B_V, Burn Vehicle; B_A, Burn Acipimox; S_A, Sham Acipimox; S_V, Sham Vehicle.

XGBoost modeling with 5-fold cross-validation was applied to each lipid class identified as significant by ANOVA to assess their discriminative power for group separation. The lipid class with the highest AUC varied by comparison. For the burn acipimox *vs.* burn vehicle comparison, LPC showed the highest AUC of 0.804. Additionally, TAG demonstrated an AUC of 0.906 for burn vehicle *vs.* sham vehicle, and LPE performed best for sham acipimox *vs.* sham vehicle with an AUC of 0.898. AUC values for each group comparison are presented in **Figure 5A**, with full results available in **Supplementary Data Sheet 9**. All values for the top performing lipid in each group comparison were above 0.80, which is generally considered the threshold for being clinically useful in interpretation, and therefore indicate these lipids as modulatory between experimental conditions (27).

After identifying the lipid classes with the highest discriminative power in each group comparison, we analyzed the XGBoost models to determine the most influential lipid species, with non-zero importance weights indicating key modulatory roles in group classification. For the sham acipimox *vs.* sham vehicle comparison, five LPE species were important, shown in **Figure 5B**. Three contained long-chain fatty acids (LCFAs) with 14–20 carbons: LPE(16:0)-H, LPE(18:0)-H, and LPE(20:3)-H; and two contained very-long chain fatty acids (VLCFAs) with ≥22 carbons: LPE(22:4)-H and LPE(22:5)-H. In the absence of burn injury, this finding underscores the role of LPEs in revealing acipimox’s effects in the brain. The significant modulation of these LPE species by acipimox in sham groups suggests neuroprotective effects specifically linked to acipimox.

In the burn acipimox *vs.* burn vehicle comparison, nine LPC species were important contributors shown in **Figure 5C**. Six of these LPCs contained LCFAs, namely LPC(14:0)+AcO, LPC(16:1)+AcO, LPC(18:0)+AcO, LPC(18:1)+AcO, LPC(20:0)+AcO., and LPC(20:3)+AcO.LPC(20:3). The remaining three LPCs contained VLCFAs: LPC(22:4)+AcO, LPC(22:5)+AcO, and LPC(22:6)+AcO. The activation of these LPCs following burn injury and their reduction with acipimox treatment demonstrates both LPCs role in burn injury, and acipimox’s modulatory capacity for neuronal damage. LPC reductions can attenuate pro-inflammatory signaling pathways, underscoring the therapeutic potential of acipimox in managing burn-induced inflammation.

In the burn acipimox *vs*. sham acipimox comparison, seven PG species, all containing LCFAs, emerged as important: PG(14:0/20:1)-H, PG(18:0/18:3)-H, PG(18:0/20:1)-H, PG(18:1/20:4)-H, PG(18:2/16:1)-H, PG(20:0/20:2)-H, and PG(20:0/20:4)-H. Important PG species are shown in **Figure 5D**. Since both groups received acipimox, the elevated levels of these specific PG species in the burn group indicate they may be linked directly to the pathological state rather than treatment effects alone. This shows that certain PGs could reflect the underlying inflammatory or metabolic changes associated with burn injury, providing potential targets for monitoring CNS disease progression and evaluating post-burn treatment response.

The burn vehicle *vs*. sham acipimox comparison highlighted six PE species, all containing LCFAs: PE(16:0/18:2)-H, PE(16:0/20:4)-H, PE(18:0/18:1)-H, PE(18:0/18:3)-H, PE(18:1/18:2)-H, and PE(18:2/20:4)-H. Important PE species are shown in **Figure 5E**. These PEs highlight burn injury’s effect on lipid-driven neuroinflammation. According to the XGBoost model, these specific PEs are critical for distinguishing between untreated burn and treated sham conditions, suggesting that burn injury elevates these neuroinflammatory lipids, while acipimox treatment may help reduce their levels.

Both in the burn vehicle vs. sham vehicle comparison, and burn acipimox *vs*. sham vehicle comparison, TAGs were identified with the highest AUC. In the burn vehicle vs. sham vehicle comparison, four TAG species containing LCFAs were key: TAG(42:2/FA18:2)+NH4, TAG(51:1/FA18:1)+NH4, TAG(53:5/FA20:4)+NH4, and TAG(56:6/FA20:5)+NH4. Important TAG species are shown in **Figure 5F**. Similarly, for the burn acipimox vs. sham vehicle comparison, two TAG species containing LCFAs were identified as important, shown in **Figure 5G**: TAG(51:1/FA18:1)+NH4 and TAG(52:6/FA18:3)+NH4. The repeated importance of TAGs across burn models highlights their critical role in the inflammatory response and lipid metabolism alterations following burn injury. Their presence in both treated and untreated burn models, compared to sham controls, underscores their potential as biomarkers of disease state post-burn and as therapeutic targets for acipimox intervention.

The identification of these lipid species rich in LCFAs (n = 28) and VLCFAs (n = 5) suggests that these fatty acids play a pivotal role in the neuroinflammatory response following burn injury. Acipimox treatment additionally appears to modulate these lipid species, indicating its influence on lipid metabolic pathways associated with inflammation and neuronal function. Full details of lipid species importance are provided in **Supplementary Data Sheet 10**.

To confirm the modulatory capacity of these identified lipid species affected by burn injury and acipimox treatment, we retrained XGBoost models using these identified lipids to classify group differences. For burn acipimox vs. burn vehicle (AUC 0.857, **Figure 5H**), burn vehicle vs. sham vehicle (AUC 0.969, **Figure 5J**), burn vehicle vs. sham acipimox (AUC 0.891, **Figure 5K**), burn acipimox vs. sham vehicle (AUC 0.964, **Figure 5L**), and burn acipimox vs. sham acipimox (AUC 0.830, **Figure 5M**), the AUC exceeded 0.80. Only in the sham acipimox vs. sham vehicle comparison (AUC 0.781, **Figure 5I**) was the AUC below 0.80. These findings suggest that, particularly in the burn models, this subgroup of identified lipid species plays a significant role in group separation and is modulated by acipimox treatment.

The normalized area ratios of this modulatory subgroup were verified, to determine trends between groups. A significantly higher normalized area ratio in the burn vehicle group was found when compared to the burn acipimox group (*p* = 0.006, difference = 0.288, 95% CI: 0.062 to 0.515), sham acipimox group (*p* < 0.001, difference = 0.541, 95% CI: 0.322 to 0.760), and sham vehicle group (*p* < 0.001, difference = 0.476, 95% CI: 0.257 to 0.695). The burn acipimox group composed of this modulatory subgroup was additionally found to be significantly elevated compared to the sham acipimox group (*p* = 0.022, difference = 0.253, 95% CI: 0.026 to 0.480), but not when compared with the sham vehicle group (*p* = 0.144, difference = 0.188, 95% CI: 0.039 to 0.414). No significant difference was found between the sham vehicle and sham acipimox groups (*p* = 0.869, difference = 0.065, 95% CI: -0.154 to 0.284). Bar plots with error bars are shown in **Figure 5N**, with details provided in **Supplementary Table 6**.

### Differential mRNA expression profiles in response to burn injury and acipimox treatment

**Figure 6:**
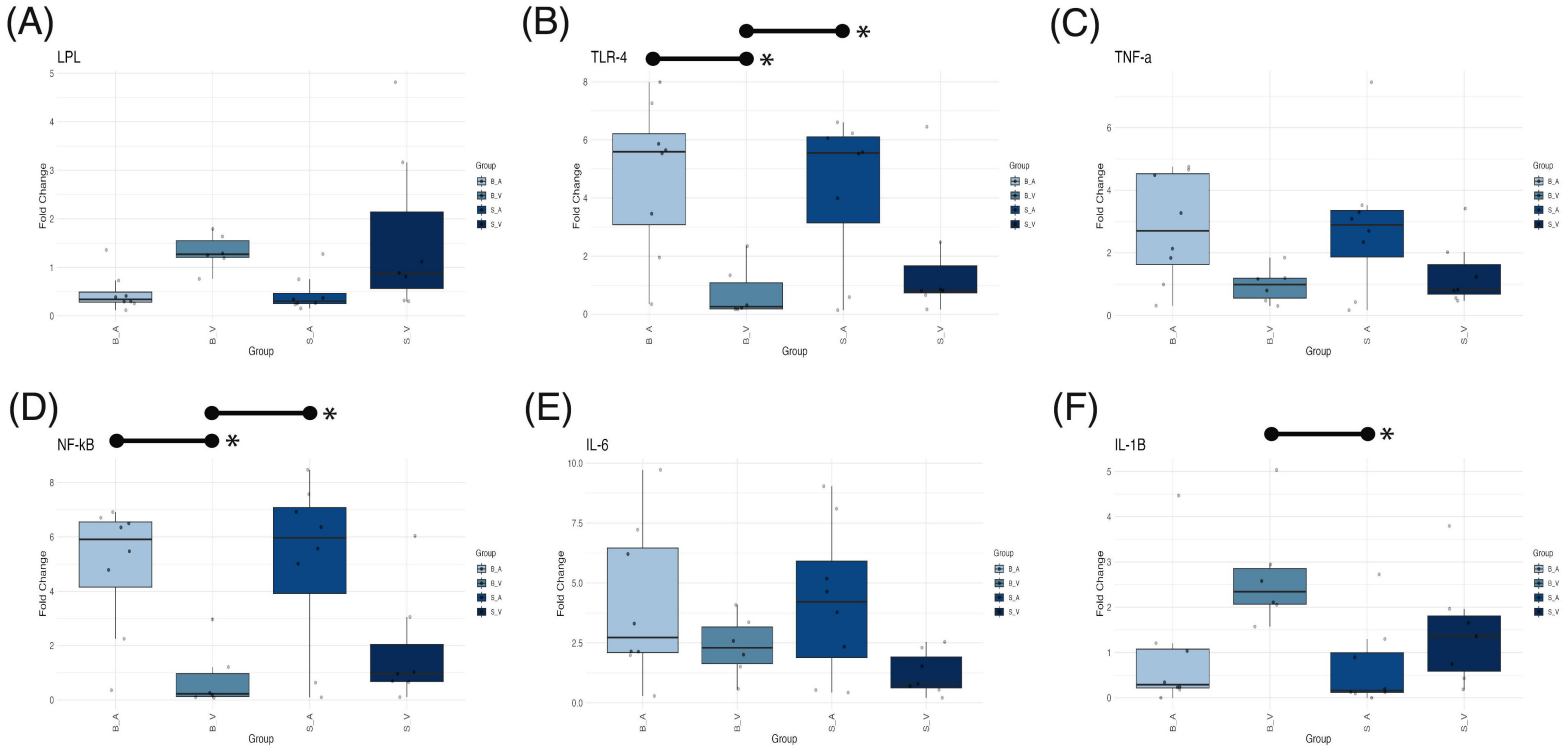
Box plots showing mRNA expression differences for **(A)** LPL, **(B)** TLR4, **(C)** TNF-α, **(D)** NF-κB, **(E)** IL-6, and **(F)** IL-1β across experimental groups. Medians are indicated by a line within each box, with box height representing the interquartile range (IQR), and whiskers extending to 1.5 times the IQR. Outliers are displayed as individual data points. Statistically significant (p < 0.05) pairwise comparisons shown by lines spanning significant groups and asterisks (*). B_V, Burn Vehicle; B_A, Burn Acipimox; S_A, Sham Acipimox; S_V, Sham Vehicle; LPL, Lipoprotein Lipase; TLR4, Toll-Like Receptor 4; TNF-α, Tumor Necrosis Factor Alpha; NF-κB, Nuclear Factor Kappa-Light-Chain-Enhancer of Activated B Cells; IL-6, Interleukin-6; IL-1β, Interleukin-1 Beta.

ANOVA testing for mRNA expression levels revealed significant group differences in fold changes for LPL (*p* = 0.041, **Figure 6A**), TLR4 (*p* = 0.006, **Figure 6B**), NF-κB (*p* = 0.0034, **Figure 6D**), and IL-1β (*p* = 0.0301, **Figure 6F**). Conversely, fold changes for TNF-α (*p* = 0.070, **Figure 6C**) and IL-6 (*p* = 0.084, **Figure 6E**) did not reach statistical significance. For IL-1β, the burn vehicle group exhibited a mean fold change of 2.720 ± SE 0.503, significantly elevated compared to the sham acipimox group (mean 0.681 ± 0.335; *p* = 0.0258, difference = 2.033, 95% CI: 0.200 to 3.868). Other pairwise comparisons for IL-1β were non-significant (*p* < 0.05) however IL-1β fold changes were higher than all other groups. These data indicate that burn injury without acipimox treatment leads to increased IL-1β expression, and that acipimox mitigates the neuroinflammatory response associated with burn injury.

In the case of NF-κB, the burn acipimox group (mean 4.920 ± 0.845) showed a significantly higher fold change than the burn vehicle group (mean 0.799 ± 0.466; *p* = 0.017, difference = 4.118, 95% CI: 0.599 to 7.637). The sham acipimox group also displayed an elevated NF-κB fold change (mean 5.08 ± 1.100) relative to the burn vehicle group (*p* = 0.013, difference = 4.280, 95% CI: 0.761 to 7.799). This trend was similarly observed for TLR4, where the burn acipimox group had a mean fold change of 4.760 ± 0.930, significantly greater than the burn vehicle group (mean 0.753 ± 0.368; *p* = 0.013, difference = 4.280, 95% CI: 0.761 to 7.799). Likewise, the sham acipimox group showed an increased TLR4 expression (mean 4.340 ± 0.912) compared to the burn vehicle group (*p* = 0.033, difference = 3.586, 95% CI: 0.225 to 6.948). This suggests that acipimox treatment, regardless of burn injury, is associated with upregulation of NF-κB and TLR4 expression levels, indicating a potential modulatory effect on inflammatory signaling pathways.

Despite the significant ANOVA result for LPL, none of the Tukey’s *post-hoc* pairwise comparisons achieved statistical significance (*p* < 0.05). The observed reduction in IL-1β, along with increased NF-κB and TLR4 in acipimox-treated groups, suggests that acipimox selectively modulates inflammation by dampening IL-1β to prevent excessive inflammation and enhancing TLR4 and NF-κB to support immune readiness. Results are provided in **Supplementary Tables 7** and **8**, with PCR data and calculations in **Supplementary Data Sheet 11**.

**Figure 7:**
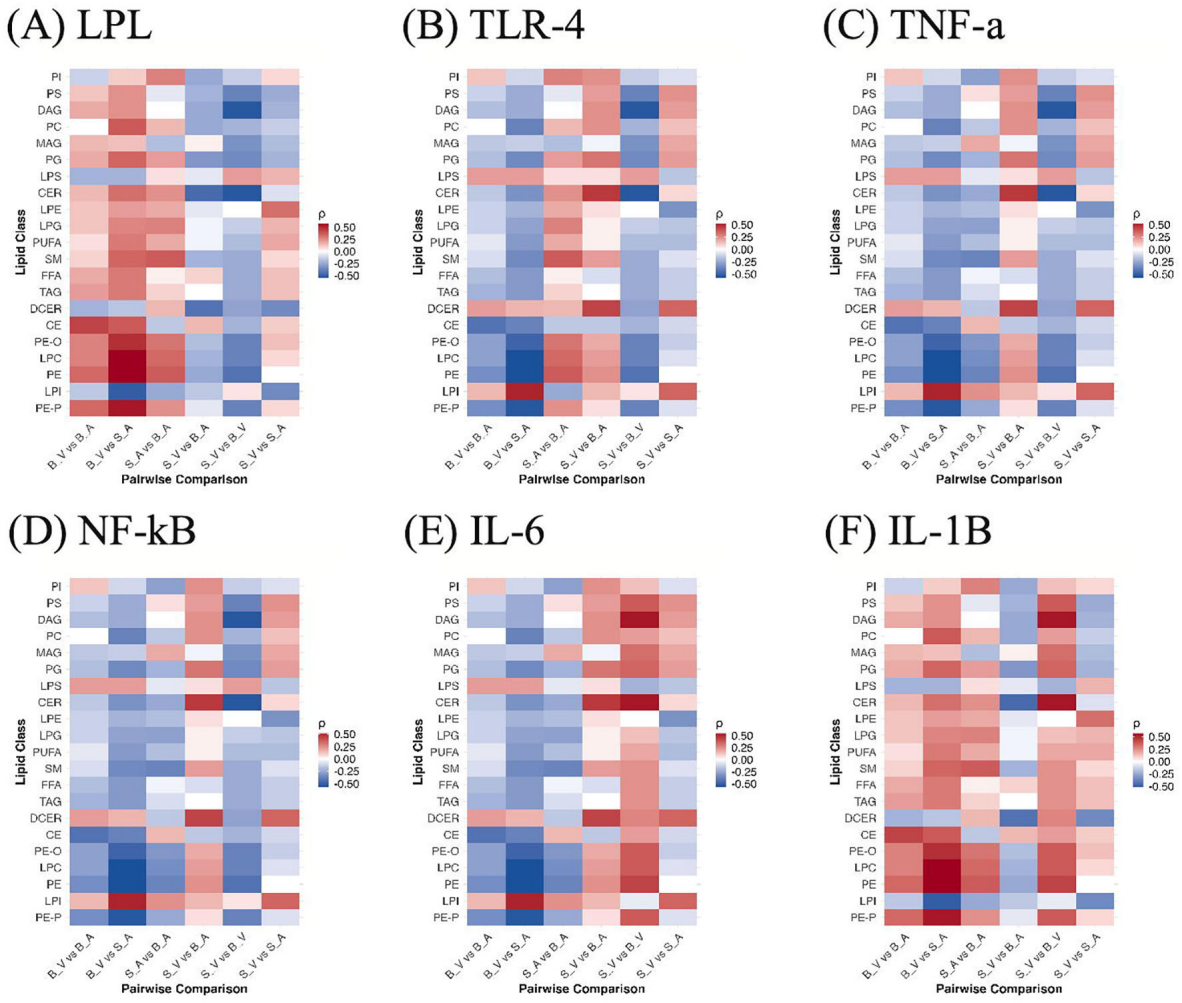
Correlations between lipid classes and cytokines across group contrasts. Heatmaps show Spearman ρ between per-sample lipid class abundances and cytokine levels for **(A)** LPL, **(B)** TLR-4, **(C)** TNF-α, **(D)** NF-κB, **(E)** IL-6, and **(F)** IL-1β across all pairwise group comparisons (S_V, B_V, S_A, B_A). Colors reflect correlation magnitude and sign. B_V, Burn Vehicle; B_A, Burn Acipimox; S_A, Sham Acipimox; S_V, Sham Vehicle. B_V, Burn Vehicle; B_A, Burn Acipimox; S_A, Sham Acipimox; S_V, Sham Vehicle; LPL, Lipoprotein Lipase; TLR4, Toll-Like Receptor 4; TNF-α, Tumor Necrosis Factor Alpha; NF-κB, Nuclear Factor Kappa-Light-Chain-Enhancer of Activated B Cells; IL-6, Interleukin-6; IL-1β, Interleukin-1 Beta.

Across burn-related contrasts, cytokine–lipid correlations showed a reproducible polarity at the class level (**Figure 7**). IL-1β (**Figure 7F**) and LPL (**Figure 7A**) were generally positively correlated with lipid classes in burn conditions, whereas TLR-4 (**Figure 7B**), TNF-α (**Figure 7C**), NF-κB (**Figure 7D**), and IL-6 (**Figure 7E**) displayed predominantly negative associations. In the untreated burn contrast (S*V vs B*V), IL-1β was positive for 18 of 21 classes, peaking for CER (ρ = +0.54) and weakest for LPS (ρ = −0.22). With acipimox treatment (S*A vs B*A), IL-1β remained positive for 16 of 21 classes, led by PE (ρ = +0.37) and least for LPI (ρ = −0.25). LPL showed a similar pattern, 16 of 21 positive in the treated contrast (top PE, ρ = +0.37), while its untreated comparison was slightly negative overall, driven by inverse correlation with CER (ρ = −0.54) despite positive relationships with several glycerophospholipids. In contrast, TNF-α, NF-κB, and TLR-4 were negative for 18 of 21 classes in S_V *vs* B*V (most negative CER, ρ = −0.54; most positive outlier LPS, ρ = +0.22), and IL-6 was negative for 16 of 21 classes in S*A vs B*A (most negative PE, ρ = −0.37). When isolating treatment within the burn cohort* (*B*V vs B_A), the same polarity held, IL-1β and LPL were again positively aligned with lipid classes, while IL-6, TNF-α, NF-κB, and TLR-4 were negative on average. None of the cytokine–lipid correlations reached statistical significance after FDR correction (q < 0.05); full correlation results with ρ and adjusted p values are provided in **Supplementary Data Sheet 12**.

## Discussion

In this study, we identified a distinctive profile of neuroinflammatory lipids that significantly increase in the prefrontal cortex following severe burn injury, and we demonstrated that acipimox treatment effectively regulates the levels of these lipids. Our findings reveal that lipid accumulation post-burn potentially exacerbates neuroinflammatory pathways, as evidenced by upregulation of pro-inflammatory markers, and acipimox, a potent anti-lipolytic agent, mitigates these changes by reducing pro-inflammatory lipid species and modulating inflammatory cytokine expression. These results suggest that targeted lipid modulation through acipimox may offer 2-deoxyglucose a promising approach to managing burn-induced neuroinflammation and its associated cognitive impairments.

Neuroinflammation following burn trauma is a complex process that is not fully understood. Upon sustaining a cutaneous burn, various mediators, including cytokines (IL-1β, IL-6, and TNFα), chemokines (CCL2, CCL5, CXCL1), reactive oxygen species (ROS), secondary messengers (NO and prostaglandins), and DAMPs are released from immune cells originating from peripheral tissue (28). The BBB traditionally acts as a safeguard, limiting the permeation of pro-inflammatory agents; however, heightened levels of interleukins, including IL-1β, may compromise this barrier (14, 29). Burn injury can increase BBB permeability through impaired tight junction protein expression, contributing to increased transcytosis (30). While the roles of cytokines and chemokines in post-burn neuroinflammation have been studied, detailed expression profiles of lipids, especially in the CNS, have not been previously described (31, 32). While IL-1β trended higher after burn and lower with acipimox, the hypothesis-relevant pairwise comparisons (BV vs SV and BA vs BV) were not statistically significant in this study. Exploratory correlation analysis further showed that IL-1β and LPL expression tended to rise in tandem with the lipid classes most elevated by burn injury, and suppressed by acipimox. We therefore interpret the transcript findings as exploratory and center our mechanistic inference on the robust lipidomic signal.

Severe burn injury induces a persistent hypermetabolic state, characterized by pronounced substrate mobilization and catabolic processes (33, 34). Adrenergic stress occurring after burns leads to the induction of a “browning” phenomenon in the subcutaneous white adipose tissue. This process notably disrupts fat metabolism by elevating lipolysis, impairing hepatic beta-oxidation, and intensifying energy substrate cycling (12, 35–37). In the context of a compromised BBB resulting from post-burn inflammation, heightened concentrations of lipid peroxidation and malondialdehyde end products can engender deleterious oxidative stress initiating apoptotic pathways (38–40). Post-burn hyperlipidemia, in combination with a permeable BBB, results in chronic neuroinflammation through activation of microglia and astrocytes, causing subsequent secretion of proinflammatory cytokines and chemokines (12, 14, 41).

Our machine learning biomarker discovery identified lipid classes with high discriminatory value for differentiating experimental groups, including LPCs, PGs, PEs, TAGs, and LPEs. While LPCs, PGs, PEs, and TAGs demonstrated significant ANOVA differences in burn-related comparisons, Previous research on neuroinflammation has characterized the roles played by LPCs and TAGs. LPCs, which are the main components of oxidized low-density lipoproteins, can accumulate and result in inflammation and neuronal cell apoptosis (42, 43). Importantly, LPCs are able to increase BBB permeability, allowing for additional pro-inflammatory cascades (44). LPCs act as DAMPs, stimulate IL-1β production, activate inflammasomes, form membrane pores leading to calcium influx, activate microglia to inflammatory states, and contribute to demyelination (45). Similarly, elevated TAG levels result in proinflammatory macrophage activation, significantly augmenting inflammatory mediators such as IL-1β and prostaglandin E2 (PGE2) (46). TAGs are crucial for energy metabolism, as their hydrolysis releases fatty acids essential for energy production or storage (46, 47). Importantly, TAGs are one of the few lipid classes able to traverse the BBB facilitating elevated levels post-burn injury (48). Furthermore, TAGs act as substrates in the synthesis of PGE2, a critical lipid mediator in neuroinflammatory processes, providing a strong positive signal for the production of IL-1β similar to LPCs (49).

A subset of the identified key modulatory lipids, PGs, and PEs, have not been previously linked to burn injury but have been studied for their integral roles in neuronal metabolic regulation and membrane composition. PGs are integral components of mitochondrial membranes in the nervous system, and alterations in their levels have been shown to contribute to neuronal degeneration and brain dysfunction (50). In our data, PG levels were elevated in burn subjects relative to sham controls; however, this elevation was not significantly attenuated by acipimox. Consistent with this, machine learning analysis identified PG species as important discriminators between burn acipimox and sham acipimox groups, suggesting that PG elevation represents a burn-associated lipid alteration that persists despite anti-lipolytic treatment. PEs constitute approximately 45% of total membrane phospholipids in the brain and are involved in membrane fluidity due to their conical shape (51). Alterations in PE levels can potentially contribute to neuroinflammatory processes (51). Because these lipids are critical for neuronal architecture and metabolism, their identification as being implicated in burn injury and modulated by acipimox treatment is vital to understanding burn-induced injury and developing therapeutic targets.

LPEs were additionally identified specifically in the sham acipimox vs. sham vehicle model, indicating acipimox-associated modulation in the absence of burn injury rather than a direct burn-associated change. This reflects potential mechanistic relevance of LPEs in CNS lipid metabolism, as identified by feature importance in the machine learning model, LPEs interact with protein networks involved in metabolic pathways, postsynaptic density, mitogen-activated protein kinase (MAPK) signaling, and the extracellular matrix (52). These interactions may underlie acipimox’s capacity to influence neuronal signaling and synaptic plasticity, potentially contributing to its neuroprotective effects and offering an additional mechanism by which it may mitigate lipid-driven neuroinflammation that warrants further exploration. Machine learning models like XGBoost evaluate lipid species based on their classification power rather than statistical significance alone. This allows for the identification of lipids such as LPE 16:0-H, which, despite not showing significant differences in ANOVA, were found to be highly relevant for distinguishing experimental groups.

We observed elevations in the mRNA expression of IL-1β in the burn injury model. Increased levels of IL-1β are linked not only to disruption of the BBB but also to increased TLR4 expression and subsequent production of chemokines and cytokines leading to neurocognitive impairment (31). IL-1β can activate microglia and astrocytes, leading to the synthesis of proinflammatory and chemotactic mediators, and perpetuate a variety of CNS diseases including multiple sclerosis, neurodegenerative diseases, traumatic brain injury, and diabetic retinopathy (53). The link between IL-1β and neurodegeneration has been validated through clinical studies showing that patients with neurodegenerative diseases exhibit higher IL-1β levels than healthy controls, which are associated with exacerbated CNS immune reactions and greater disease severity (53, 54). IL-1β mediates neuroinflammation post-injury, while TLR4 detects DAMPs and activates NF-κB, a key regulator of inflammatory genes (55, 56).

Interestingly, acipimox treatment in our study significantly reduced IL-1β levels while simultaneously upregulating NF-κB and TLR4 in acipimox-treated groups. While NF-κB is often considered pro-inflammatory, it also plays an essential role in resolving inflammation by inducing anti-inflammatory pathways and promoting cellular mechanisms like autophagy, which help to clear damaged cells and limit inflammasome activation (57, 58). This dual action of NF-κB may explain its upregulation in acipimox-treated groups, where NF-κB activation contributes to immune readiness without promoting chronic inflammation. Our findings suggest that acipimox selectively modulates inflammation by dampening IL-1β to prevent excessive inflammation and enhancing NF-κB and TLR4 activity to support a balanced immune response. This regulatory role of NF-κB highlights its importance in preventing chronic inflammation and promoting tissue homeostasis after injury (57, 58).

### Limitations and future directions

This work examined only the rat prefrontal cortex at a single post-burn time point, which limits inference about regional specificity and temporal dynamics. Elevated brain lipids may reflect peripheral lipolysis or inputs from other brain regions if the blood brain barrier is more permeable, and acipimox crosses this barrier so effects may be both peripheral and central (59). Future studies should sample additional regions that support cognition such as the hippocampus and amygdala and should quantify lipid mediator expression across multiple time points in larger cohorts. It will also be important to define the direct mechanisms by which acipimox modulates lipid metabolism and inflammatory signaling within the central nervous system, and to link treatment to behavioral and cognitive outcomes to connect lipid modulation with functional change.

Concurrent systemic interventions in animals used in this study may introduce potential confounding. Subsets received leucine with insulin or PBS on day 7, and all animals received 2-deoxyglucose and 2H5 phenylalanine before euthanasia. Insulin can alter central lipid metabolism and inflammatory signaling and 2-deoxyglucose can influence neuroinflammation (60, 61). Groups were randomized and balanced, yet differential responses between burn and control animals were not stratified or adjusted, so residual confounding cannot be excluded. Interpretation of lipidomics should also remain cautious. Findings are associative and we did not measure neuropathology, protein level cytokines, or behavior, and several lipid classes that rose after burn were not reduced by acipimox. Some model important features arose from sham contrasts and did not reach ANOVA significance in burn related comparisons, cross validated AUC values above 0.80 indicate discrimination rather than causation, and the PLS DA permutation test was not significant (p=0.13). A subset of lipid shifts may be compensatory rather than harmful, and acipimox may act through GPR109A signaling independent of lipid lowering. Methodologically, targeted LC MS reported normalized area ratios rather than absolute molar concentrations, which is suitable for group comparisons but can favor well ionizing species and underestimate low abundance lipids. Stronger tests will pair lipidomics with functional readouts, spatial lipid mapping, longitudinal sampling, absolute quantitation, and targeted perturbations in larger cohorts that are free of concurrent systemic interventions.

Three analytic points merit specific attention. First, the reference gene CycA varied with treatment in RT qPCR, and the 2^-ΔΔCt method assumes a stable reference, so fold changes may be inflated or deflated in acipimox groups. Replication with multiple validated reference genes selected by standard stability algorithms or alternative normalization, plus orthogonal confirmation at the protein or cellular level in larger cohorts without concurrent interventions, will be important to determine whether acipimox truly attenuates neuroinflammation. Second, the machine learning analyses were discovery oriented without a blinded holdout set or external cohort, and the modulatory subset was defined on the full dataset before refitting, which can introduce selection bias. Features should be treated as candidates that require future confirmation using fully nested cross validation with all preprocessing and feature selection inside training folds, followed by testing in independent validation sets that span batches and time points. Finally, the study was powered for large omnibus effects and large pairwise differences (Cohen’s f=0.64, partial η²=0.29; d=1.75–1.80), so smaller effects may have been missed, which reinforces the need for larger animal cohorts.

To better understand cytokine dynamics, future studies should investigate the timing and regulation of key inflammatory mediators following burn trauma. Although IL-6 and TNF-α are well-established drivers of neuroinflammation and neurodegeneration (62), their muted response in our model suggests delayed activation or suppression by early IL-1β signaling. Early targeting of IL-1β, along with its lipid-linked mediators such as PGE_2_, may offer therapeutic benefit, especially given that endogenous PGE_2_ promotes IL-1β production (49) while attenuating TNF-α (49). Exploring these interactions, such as with IL-1β or COX-2/PGE_2_ inhibitors, could clarify whether selective modulation of this network improves outcomes. In parallel, future studies should correlate additional neuroinflammatory endpoints, including microglial activation and blood levels of neuron-specific enolase, to capture cellular and functional aspects of CNS injury.

## Conclusion

In this study, we have characterized a CNS lipid profile associated with burn injury, which is modulated through acipimox treatment. Our findings suggest that acipimox has the potential to mitigate burn-induced neuroinflammation by modulating specific lipid species and inflammatory pathways, offering a promising therapeutic approach for managing the neurological complications associated with severe burns.

## Data Availability

The original contributions presented in the study are included in the article/**Supplementary Material**. Further inquiries can be directed to the corresponding author.

## Supplementary material

SUPPLEMENTARY FIGURE 1 Results of data normalization using Relative Standard Deviation (RSD), Interquartile Range (IQR) filtering, log transformation, and standard deviation scaling.
