# Supplementary figures and images for: Altered lipid profiles in the prefrontal cortex are associated with neuroinflammation after severe burn injury
Source: Front Immunol. 2025 Dec 1;16:1709256. doi: 10.3389/fimmu.2025.1709256 (PMC12702713; doi:10.3389/fimmu.2025.1709256)

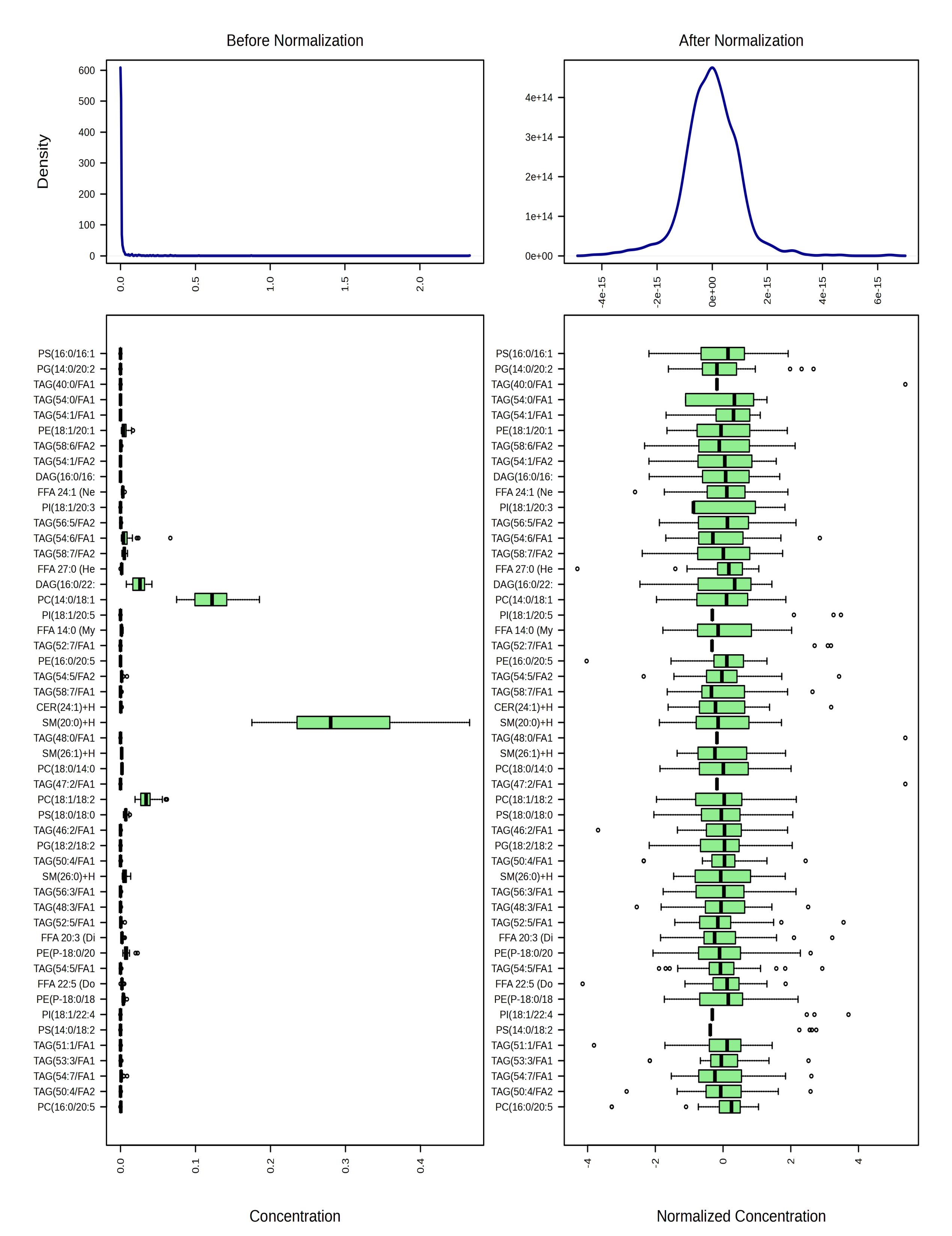

Supplement: SUPPLEMENTARY FIGURE 1 — Results of data normalization using Relative Standard Deviation (RSD), Interquartile Range (IQR) filtering, log transformation, and standard deviation scaling. [file Image1.jpeg]
