# Supplementary material for: Altered lipid profiles in the prefrontal cortex are associated with neuroinflammation after severe burn injury
Source: Front Immunol. 2025 Dec 1;16:1709256. doi: 10.3389/fimmu.2025.1709256 (PMC12702713; doi:10.3389/fimmu.2025.1709256)
Supplement: Supplementary file 14 [file Supplementaryfile1.docx]

**Supplemental Table 1.** PCR primer set sequences used in this study.
*Abbreviations*: IL-1β, Interleukin-1 Beta; IL-6, Interleukin-6; LPL, Lipoprotein Lipase; NF-κB, Nuclear Factor Kappa-Light-Chain-Enhancer of Activated B Cells; TNF-α, Tumor Necrosis Factor Alpha; TLR-4, Toll-Like Receptor 4; Cyc-A, Cyclophilin A; F, Forward; R, Reverse.

| **Primer** | **Sequence** |
| --- | --- |
| **IL-1b-F** | CCT TTG CCA AAT CTG CTC TC |
| **IL1-b-R** | TGC TCT GGA GTA GGG ATG CT |
| **Il-6-F** | TGA AGA CCG GCT ACT GTG GAA GAG AC |
| **Il-6-R** | TTG GGG TGA AAG TGA GAC GGA GCA G |
| **LPL- F** | TCG ACA TCT ACA AGC AGG AA |
| **LPL-R** | CTG TCC TTG GAA GAT GCT TT |
| **NFkb-F** | TTC CCT GAC CAG TTC TTA GTG C |
| **NFkb-R** | CTG CGG ACT ATC TTC AGC TGA T |
| **TNF-aF** | TCA TCT TCT CAA AAC TCG AGT GAC A |
| **TNF-aR** | TGT CTA AGT ACT TGG GCA GGT TGA |
| **TLR-4 F** | GAT TGC TCA GAC ATG GCA GTT TC |
| **TLR-4 R** | CAC TCG AGG TAG GTG TTT CTG CTA A |
| **Cyc-A F** | ACG GAC CAG AGC GAA AGC AT |
| **Cyc-A R** | TGT CAA TCC TGT CCG TGT CC |

**Supplemental Table 2:** Lipid class composition and frequency distribution within the four clusters identified in hierarchical clustering analysis. Each cluster lists lipid classes, frequencies, and representative lipid species. *Abbreviations*: AA, Arachidonic Acid; CER, Ceramide; DAG, Diacylglycerol; PC, Phosphatidylcholine; PE, Phosphatidylethanolamine; PE-P, Phosphatidylethanolamine plasmalogen; PG, Phosphatidylglycerol; PI, Phosphatidylinositol; PS, Phosphatidylserine; SM, Sphingomyelin; TAG, Triacylglycerol.

| Cluster | Lipid Class | Frequency | Lipid Species |
| --- | --- | --- | --- |
| 1 | DAG | 3 | DAG(16:1/18:2), DAG(18:0/18:3), DAG(18:2/18:3) |
|  | PG | 1 | PG(18:2/16:1)-H |
|  | PI | 1 | PI(18:0/20:1)-H |
|  | TAG | 1 | TAG(51:1/FA18:1) |
| 2 | DAG | 1 | DAG(14:0/20:0) |
|  | PC | 2 | PC(14:0/18:3), PC(16:0/18:2) |
|  | PE | 8 | PE(16:0/18:2)-H, PE(18:0/18:2)-H, PE(18:1/18:2)-H, PE(18:2/18:2)-H, PE(18:2/20:2)-H, PE(P-18:1/18:2)-H, PE(P-18:1/18:3)-H, PE(P-18:2/18:2)-H |
|  | PG | 5 | PG(14:0/18:2)-H, PG(16:0/18:2)-H, PG(16:0/20:2)-H, PG(18:0/18:2)-H, PG(18:0/20:2)-H |
|  | PS | 2 | PS(18:0/18:2)-H, PS(20:0/18:2)-H |
|  | SM | 2 | SM(20:0), SM(20:1) |
| 3 | CER | 3 | CER(20:1), CER(26:0), CER(26:1) |
|  | DAG | 7 | DAG(14:0/18:1), DAG(16:0/18:0), DAG(16:0/18:3), DAG(16:0/20:5), DAG(16:1/18:1), DAG(16:1/20:2), DAG(16:1/20:4) |
|  | PG | 2 | PG(20:0/20:4)-H, PG(20:0/22:4)-H |
|  | TAG | 2 | TAG(51:2/FA18:1), TAG(52:6/FA18:1) |
| 4 | CE | 1 | CE(18:1) |
|  | CER | 3 | CER(22:1), CER(24:0), CER(24:1) |
|  | DAG | 5 | DAG(14:0/20:4), DAG(16:0/18:1), DAG(16:0/18:2), DAG(16:0/20:4), DAG(18:0/18:2) |
|  | PC | 1 | PC(18:1/20:2) |

**Supplemental Table 3:** Cross-validation results for the PLS-DA model with 1-5 components, showing accuracy, R², and Q² values for each component. *Abbreviations*: PLS-DA, Partial Least Squares-Discriminant Analysis; R², determination coefficient; Q², predictive ability.

| **Measure** | **1 comps** | **2 comps** | **3 comps** | **4 comps** | **5 comps** |
| --- | --- | --- | --- | --- | --- |
| **Accuracy** | 0.20857 | 0.29905 | 0.33238 | 0.37238 | 0.36762 |
| **R2** | 0.52406 | 0.82811 | 0.95 | 0.98108 | 0.99647 |
| **Q2** | -0.11444 | 0.07808 | 0.17967 | 0.11805 | 0.092439 |

**Supplemental Table 4:** Mean ± SEM values for lipid class profiles across experimental groups with one-way ANOVA p-values. Statistically significant differences (p < 0.05) across groups are marked by an asterisk (*). *Abbreviations*: CE, Cholesteryl Ester; CER, Ceramide; DAG, Diacylglycerol; DCER, Dihydroceramide; FFA, Free Fatty Acid; LPC, Lysophosphatidylcholine; LPE, Lysophosphatidylethanolamine; LPG, Lysophosphatidylglycerol; LPI, Lysophosphatidylinositol; MAG, Monoacylglycerol; PC, Phosphatidylcholine; PE, Phosphatidylethanolamine; PE-O, Phosphatidylethanolamine O-alkyl; PE-P, Phosphatidylethanolamine plasmalogen; PG, Phosphatidylglycerol; PI, Phosphatidylinositol; PS, Phosphatidylserine; SM, Sphingomyelin; TAG, Triacylglycerol; PUFA, Polyunsaturated Fatty Acid.

| **Lipid** | **BA (Mean ± SEM)** | **BV (Mean ± SEM)** | **SA (Mean ± SEM)** | **SV (Mean ± SEM)** | **ANOVA p-values** |
| --- | --- | --- | --- | --- | --- |
| **SM** | 0.149 ± 0.107 | 0.313 ± 0.106 | -0.348 ± 0.087 | -0.096 ± 0.097 | <0.001* |
| **CE** | -0.151 ± 0.170 | 0.235 ± 0.177 | -0.053 ± 0.162 | -0.051 ± 0.197 | 0.502 |
| **CER** | 0.145 ± 0.096 | 0.235 ± 0.104 | -0.117 ± 0.108 | -0.246 ± 0.092 | 0.004* |
| **DCER** | 0.172 ± 0.095 | -0.011 ± 0.134 | 0.121 ± 0.137 | -0.261 ± 0.120 | 0.093 |
| **TAG** | -0.078 ± 0.015 | 0.258 ± 0.018 | -0.129 ± 0.017 | -0.061 ± 0.018 | <0.001* |
| **DAG** | -0.031 ± 0.054 | 0.229 ± 0.045 | -0.017 ± 0.052 | -0.186 ± 0.047 | <0.001* |
| **MAG** | -0.149 ± 0.093 | 0.150 ± 0.087 | 0.103 ± 0.080 | -0.122 ± 0.082 | 0.035* |
| **LPC** | 0.003 ± 0.093 | 0.465 ± 0.088 | -0.309 ± 0.084 | -0.159 ± 0.097 | <0.001* |
| **PC** | 0.100 ± 0.042 | 0.110 ± 0.040 | -0.055 ± 0.035 | -0.143 ± 0.042 | <0.001* |
| **LPE** | 0.056 ± 0.089 | 0.110 ± 0.108 | -0.171 ± 0.089 | 0.012 ± 0.088 | 0.215 |
| **PE** | 0.044 ± 0.040 | 0.403 ± 0.043 | -0.271 ± 0.036 | -0.171 ± 0.051 | <0.001* |
| **PE-O** | -0.003 ± 0.061 | 0.446 ± 0.069 | -0.334 ± 0.056 | -0.109 ± 0.072 | <0.001* |
| **PE-P** | -0.068 ± 0.053 | 0.513 ± 0.049 | -0.325 ± 0.038 | -0.129 ± 0.053 | <0.001* |
| **LPG** | -0.001 ± 0.085 | 0.225 ± 0.103 | -0.296 ± 0.082 | 0.071 ± 0.086 | <0.001* |
| **PG** | 0.075 ± 0.042 | 0.149 ± 0.039 | -0.031 ± 0.036 | -0.183 ± 0.042 | <0.001* |
| **LPI** | 0.046 ± 0.155 | -0.113 ± 0.120 | 0.166 ± 0.144 | -0.093 ± 0.117 | 0.472 |
| **PI** | 0.023 ± 0.047 | 0.002 ± 0.045 | -0.030 ± 0.045 | 0.009 ± 0.047 | 0.869 |
| **LPS** | 0.171 ± 0.269 | -0.172 ± 0.080 | -0.085 ± 0.150 | 0.108 ± 0.275 | 0.649 |
| **PS** | 0.000 ± 0.048 | 0.107 ± 0.045 | 0.005 ± 0.040 | -0.112 ± 0.044 | 0.009* |
| **FFA** | -0.082 ± 0.059 | 0.155 ± 0.069 | -0.095 ± 0.045 | 0.012 ± 0.062 | 0.0160* |
| **PUFA** | 0.007 ± 0.165 | 0.165 ± 0.196 | -0.243 ± 0.101 | 0.071 ± 0.156 | 0.361 |

**Supplemental Table 5:** One-way ANOVA and Tukey post-hoc pairwise comparisons for lipid classes across groups (Burn Vehicle, Burn Acipimox, Sham Acipimox, Sham Vehicle), with ANOVA p-values and pairwise p-values with mean differences and 95% confidence intervals (lower bound [LB] to upper bound [UB]). Statistically significant comparisons (p < 0.05) are marked by an asterisk (*). *Abbreviations*: CER, Ceramides; DAG, Diacylglycerols; FFA, Free Fatty Acids; LPC, Lysophosphatidylcholines; LPG, Lysophosphatidylglycerols; MAG, Monoacylglycerols; PC, Phosphatidylcholines; PE, Phosphatidylethanolamines; PE-O, Ether-linked Phosphatidylethanolamines; PE-P, Phosphatidylethanolamine plasmalogens; PG, Phosphatidylglycerols; PS, Phosphatidylserines; SM, Sphingomyelins; TAG, Triacylglycerols.

| Lipid  Class | ANOVA *p-value* | BV *vs.* B A  *p-value* (difference, LB to UB) | SA *vs.* BA  *p-value* (difference, LB to UB) | SV *vs.* BA  *p-value* (difference, LB to UB) | SA *vs.* BV  *p-value* (difference, LB to UB) | SV *vs.* BV  *p-value* (difference, LB to UB) | SV *vs.* SA  *p-value* (difference, LB to UB) |
| --- | --- | --- | --- | --- | --- | --- | --- |
| CER | 0.004* | 0.834 (0.116, -0.240 to 0.473) | 0.323 (-0.235, -0.592 to 0.121) | 0.043* (-0.364, -0.721 to -0.008) | 0.055 (-0.352, -0.708 to 0.005) | 0.003* (-0.481, -0.837 to -0.124) | 0.787 (-0.129, -0.485 to 0.228) |
| DAG | <0.001* | 0.002* (0.242, 0.067 to 0.417) | 1.000 (-0.004, -0.179 to 0.171) | 0.054 (-0.173, -0.348 to 0.002) | 0.002* (-0.246, -0.420 to -0.071) | <0.001* (-0.415, -0.590 to -0.240) | 0.062 (-0.169, -0.344 to 0.006) |
| FFA | 0.016* | 0.036* (0.220, 0.010 to 0.431) | 0.983 (-0.030, -0.240 to 0.180) | 0.782 (0.077, -0.133 to 0.287) | 0.012* (-0.250, -0.461 to -0.040) | 0.296 (-0.143, -0.354 to 0.067) | 0.557 (0.107, -0.103 to 0.317) |
| LPC | <0.001* | <0.001* (0.493, 0.172 to 0.813) | 0.109 (-0.281, -0.602 to 0.040) | 0.715 (-0.132, -0.452 to 0.189) | <0.001* (-0.774, -1.094 to -0.453) | <0.001* (-0.624, -0.945 to -0.304) | 0.625 (0.150, -0.171 to 0.470) |
| LPG | <0.001* | 0.215 (0.239, -0.080 to 0.557) | 0.102 (-0.283, -0.601 to 0.036) | 0.903 (0.085, -0.234 to 0.403) | <0.001* (-0.521, -0.839 to -0.203) | 0.596 (-0.154, -0.472 to 0.164) | 0.016* (0.367, 0.049 to 0.685) |
| MAG | 0.035* | 0.059 (0.294, -0.008 to 0.596) | 0.152 (0.247, -0.055 to 0.549) | 0.997 (0.023, -0.279 to 0.325) | 0.978 (-0.047, -0.349 to 0.255) | 0.095 (-0.271, -0.573 to 0.030) | 0.223 (-0.224, -0.526 to 0.078) |
| PC | <0.001* | 0.984 (0.020, -0.121 to 0.160) | 0.039* (-0.146, -0.286 to -0.005) | <0.001* (-0.233, -0.373 to -0.092) | 0.013* (-0.165, -0.306 to -0.025) | <0.001* (-0.253, -0.393 to -0.112) | 0.381 (-0.087, -0.228 to 0.053) |
| PE | <0.001* | <0.001* (0.376, 0.224 to 0.529) | <0.001* (-0.297, -0.450 to -0.144) | 0.005* (-0.197, -0.350 to -0.044) | <0.001* (-0.674, -0.827 to -0.521) | <0.001* (-0.573, -0.726 to -0.420) | 0.329 (0.100, -0.052 to 0.253) |
| PE-O | <0.001* | <0.001* (0.448, 0.217 to 0.678) | 0.001* (-0.332, -0.562 to -0.101) | 0.627 (-0.107, -0.338 to 0.123) | <0.001* (-0.779, -1.010 to -0.549) | <0.001* (-0.555, -0.786 to -0.325) | 0.060 (0.224, -0.006 to 0.455) |
| PE-P | <0.001* | <0.001* (0.571, 0.401 to 0.742) | <0.001* (-0.266, -0.437 to -0.095) | 0.716 (-0.070, -0.241 to 0.101) | <0.001* (-0.838, -1.009 to -0.667) | <0.001* (-0.642, -0.812 to -0.471) | 0.017* (0.196, 0.025 to 0.367) |
| PG | <0.001* | 0.466 (0.079, -0.061 to 0.220) | 0.254 (-0.100, -0.241 to 0.040) | <0.001* (-0.253, -0.393 to -0.113) | 0.005* (-0.180, -0.320 to -0.040) | <0.001* (-0.332, -0.472 to -0.192) | 0.027* (-0.152, -0.293 to -0.012) |
| PS | 0.009* | 0.384 (0.096, -0.059 to 0.252) | 0.999 (-0.005, -0.161 to 0.150) | 0.182 (-0.122, -0.278 to 0.034) | 0.334 (-0.102, -0.257 to 0.054) | 0.002* (-0.218, -0.374 to -0.063) | 0.216 (-0.117, -0.272 to 0.039) |
| SM | <0.001* | 0.643 (0.160, -0.191 to 0.511) | <0.001* (-0.502, -0.853 to -0.150) | 0.262 (-0.249, -0.600 to 0.102) | <0.001* (-0.662, -1.013 to -0.310) | 0.015* (-0.409, -0.760 to -0.058) | 0.249 (0.253, -0.099 to 0.604) |
| TAG | <0.001* | <0.001* (0.293, 0.232 to 0.354) | <0.001* (-0.095, -0.155 to -0.034) | 0.680 (-0.026, -0.087 to 0.034) | <0.001* (-0.387, -0.448 to -0.326) | <0.001* (-0.319, -0.380 to -0.258) | 0.021* (0.068, 0.007 to 0.129) |

**Supplemental Table 6:** One-way ANOVA and Tukey post-hoc pairwise comparisons of normalized area ratios for modulatory lipids identified through machine learning biomarker discovery. Mean ± SEM values for each group, with statistically significant differences (p < 0.05) marked by an asterisk (*). *Abbreviations*: BV, Burn Vehicle; BA, Burn Acipimox; SV, Sham Vehicle; SA, Sham Acipimox.

| **ANOVA *p-value*** | **Group Comparison (1 *vs.* 2)** | **Group 1 (Mean ± SEM)** | **Group 2 (Mean ± SEM)** | ***p-value* (Difference, LB to UB)** |
| --- | --- | --- | --- | --- |
| <0.001* | **BV vs. BA** | 0.328 ± 0.054 | 0.0394 ± 0.054 | 0.006* (0.288, 0.062 to 0.515) |
|  | **SA vs. BA** | -0.214 ± 0.062 | 0.0394 ± 0.054 | 0.022* (-0.253, -0.480 to -0.026) |
|  | **SV vs. BA** | -0.148 ± 0.064 | 0.0394 ± 0.054 | 0.144 (-0.188, -0.414 to 0.039) |
|  | **SA vs. BV** | -0.214 ± 0.062 | 0.328 ± 0.054 | <0.001* (-0.541, -0.760 to -0.322) |
|  | **SV vs. BV** | -0.148 ± 0.064 | 0.328 ± 0.054 | <0.001* (-0.476, -0.695 to -0.257) |
|  | **SV vs. SA** | -0.148 ± 0.064 | -0.214 ± 0.062 | 0.869 (0.065, -0.154 to 0.284) |

**Supplemental Table 7:** Fold changes for cytokines between groups, reported as mean ± SEM for each group. *Abbreviations*: BV, Burn Vehicle; BA, Burn Acipimox; SV, Sham Vehicle; SA, Sham Acipimox.

| **Cytokine** | **BV (Mean ± SEM)** | **BA (Mean ± SEM)** | **SV (Mean ± SEM)** | **SA (Mean ± SEM)** |
| --- | --- | --- | --- | --- |
| **IL-1β** | 2.72 ± 0.503 | 0.961 ± 0.523 | 1.45 ± 0.462 | 0.681 ± 0.335 |
| **IL-6** | 2.36 ± 0.517 | 4.13 ± 1.14 | 1.23 ± 0.344 | 4.26 ± 1.13 |
| **NF-κB** | 0.799 ± 0.466 | 4.92 ± 0.845 | 1.79 ± 0.791 | 5.08 ± 1.10 |
| **TLR-4** | 0.753 ± 0.368 | 4.76 ± 0.930 | 1.74 ± 0.830 | 4.34 ± 0.912 |
| **TNF-α** | 0.964 ± 0.230 | 2.80 ± 0.615 | 1.33 ± 0.400 | 2.87 ± 0.793 |
| **LPL** | 1.32 ± 0.148 | 0.481 ± 0.140 | 1.63 ± 0.645 | 0.457 ± 0.133 |

**Supplemental Table 8:** One-way ANOVA and Tukey post-hoc pairwise comparisons of normalized area ratios for modulatory lipids identified through biomarker discovery. Values are reported as mean ± SEM for each group, with statistically significant differences (p < 0.05) marked by an asterisk (*).

| **Cytokine** | **ANOVA *p-value*** | **BV *vs.* B A**  ***p-value* (difference, LB to UB)** | **SA *vs.* BA**  ***p-value* (difference, LB to UB)** | **SV *vs.* BA**  ***p-value* (difference, LB to UB)** | **SA *vs.* BV**  ***p-value* (difference, LB to UB)** | **SV *vs.* BV**  ***p-value* (difference, LB to UB)** | **SV *vs.* SA**  ***p-value* (difference, LB to UB)** |
| --- | --- | --- | --- | --- | --- | --- | --- |
| **IL-1β** | 0.030* | 0.194 (1.754, -0.080 to 3.588) | 0.968 (-0.280, -1.978 to 1.418) | 0.968 (0.486, -1.272 to 2.244) | 0.155 (-2.034, -3.868 to -0.200) | 0.554 (-1.268, -3.157 to 0.622) | 0.950 (0.766, -0.992 to 2.524) |
| **IL-6** | 0.084 | 0.850 (-1.770, -5.494 to 1.953) | 1.000 (0.130, -3.317 to 3.578) | 0.427 (-2.895, -6.463 to 0.673) | 0.850 (1.901, -1.823 to 5.624) | 1.000 (-1.125, -4.960 to 2.711) | 0.427 (-3.025, -6.593 to 0.543) |
| **NF-κB** | 0.003* | 0.052 (-4.118, -7.637 to -0.599) | 1.000 (0.162, -3.095 to 3.420) | 0.114 (-3.126, -6.498 to 0.247) | 0.052 (4.280, 0.761 to 7.799) | 1.000 (0.992, -2.633 to 4.617) | 0.114 (-3.288, -6.660 to 0.084) |
| **TLR-4** | 0.006* | 0.092 (-4.003, -7.364 to -0.642) | 0.983 (-0.417, -3.529 to 2.695) | 0.146 (-3.013, -6.234 to 0.209) | 0.100 (3.586, 0.225 to 6.948) | 0.983 (0.991, -2.472 to 4.453) | 0.219 (-2.596, -5.817 to 0.625) |
| **TNF-α** | 0.070 | 0.466 (-1.839, -4.228 to 0.550) | 1.000 (0.071, -2.141 to 2.282) | 0.466 (-1.473, -3.762 to 0.816) | 0.466 (1.909, -0.479 to 4.298) | 1.000 (0.365, -2.096 to 2.826) | 0.466 (-1.544, -3.833 to 0.745) |
| **LPL** | 0.041* | 0.496 (0.840, -0.496 to 2.175) | 1.000 (-0.024, -1.261 to 1.213) | 0.269 (1.150, -0.130 to 2.430) | 0.496 (-0.864, -2.199 to 0.472) | 1.000 (0.310, -1.066 to 1.687) | 0.269 (1.174, -0.106 to 2.454) |
